# Short-Term Supplementation of Sodium Nitrate vs. Sodium Chloride Increases Homoarginine Synthesis in Young Men Independent of Exercise

**DOI:** 10.3390/ijms231810649

**Published:** 2022-09-13

**Authors:** Dimitrios Tsikas, Norbert Maassen, Antonie Thorns, Armin Finkel, Moritz Lützow, Magdalena Aleksandra Röhrig, Larissa Sarah Blau, Laurianne Dimina, François Mariotti, Bibiana Beckmann, Vladimir Shushakov, Mirja Jantz

**Affiliations:** 1 Core Unit Proteomics, Institute of Toxicology, Hannover Medical School, 30623 Hannover, Germany; 2 Institute of Sport Medicine, Hannover Medical School, 30623 Hannover, Germany; 3 Institute of Sport Science, Leibniz University Hannover, 30167 Hannover, Germany; 4 AgroParisTech, INRAE, UMR PNCA, Université Paris-Saclay, 75005 Paris, France

**Keywords:** amino acids, guanidinoacetate, homoarginine, inorganic nitrate, malondialdehyde, oxidative stress, power, sports, supplementation

## Abstract

The aim of the study was to investigate the effects of short-term oral administration of inorganic nitrate (NaNO_3_; *n* = 8) or placebo (NaCl; *n* = 9) (each 0.1 mmol/kg body weight/d for 9 days) on plasma amino acids, creatinine, and oxidative stress in healthy young men. At baseline, the plasma concentrations of amino acids did not differ between the groups. At the end of the study, the plasma concentrations of homoarginine (hArg; by 24%, *p* = 0.0001), citrulline and ornithine (Cit/Orn; by 16%, *p* = 0.015), and glutamine/glutamate (Gln/Glu; by 6%, *p* = 0.0003) were higher in the NaNO_3_ group compared to the NaCl group. The plasma concentrations of sarcosine (Sarc; by 28%, *p* < 0.0001), tyrosine (by 14%, *p* = 0.0051), phenylalanine (by 8%, *p* = 0.0026), and tryptophan (by 8%, *p* = 0.0047) were lower in the NaNO_3_ group compared to the NaCl group. These results suggest that nitrate administration affects amino-acid metabolism. The arginine/glycine amidinotransferase (AGAT) catalyzes two reactions: (1) the formation of l-homoarginine (hArg) and l-ornithine (Orn) from l-arginine (Arg) and l-lysine (Lys): Arg + Lys <−> hArg + Orn, with equilibrium constant *K*_harg_; (2) the formation of guanidinoacetate (GAA) and Orn from Arg and glycine (Gly): Arg + Gly <−> GAA + Orn, with equilibrium constant *K*_gaa_. The plasma *K*_gaa_/*K*_hArg_ ratio was lower in the NaNO_3_ group compared to the NaCl group (1.57 vs. 2.02, *p* = 0.0034). Our study suggests that supplementation of inorganic nitrate increases the AGAT-catalyzed synthesis of hArg and decreases the *N*-methyltransferase-catalyzed synthesis of GAA, the precursor of creatine. To our knowledge, this is the first study to demonstrate elevation of hArg synthesis by inorganic nitrate supplementation. Remarkably, an increase of 24% corresponds to the synthesis capacity of one kidney in healthy humans. Differences in the association between plasma concentrations of amino acids in the NaNO_3_ and NaCl groups suggest changes in amino-acid homeostasis. Plasma concentrations of the oxidative stress marker malondialdehyde (MDA) did not change after supplementation of NaNO_3_ or NaCl over the whole exercise time range. Plasma nitrite concentration turned out to be a more discriminant marker of NaNO_3_ ingestion than plasma nitrate (area under the receiver operating characteristic curve: 0.951 vs. 0.866, *p* < 0.0001 each).

## 1. Introduction

**Scheme 1:**
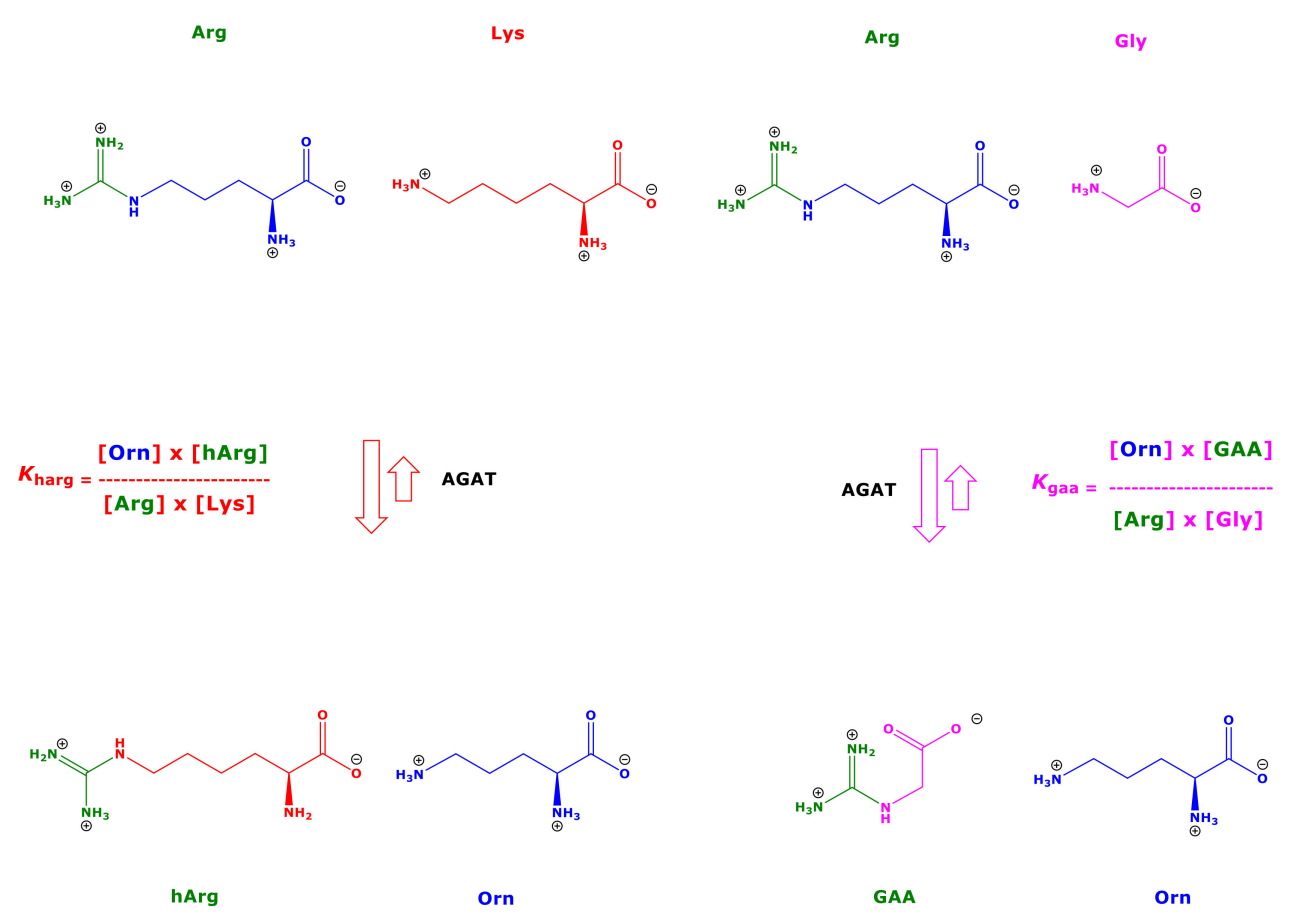
Simplified schematic of the two main reactions catalyzed by l-arginine/glycine amidinotransferase (AGAT) and formulae to calculate the equilibrium constants *K*_harg_ and *K*_gaa_ with respect to homoarginine (hArg) and guanidinoacetate (GAA) formation, respectively. The equilibrium concentrations of the involved amino acids are given in square brackets. The “forward” reactions are assumed to be preferred to the “reverse” reactions.

Amino acids are not only the building blocks of proteins; they are also involved in numerous pathways and play important roles in living organisms. In one of these pathways, l-arginine/glycine amidinotransferase (AGAT; EC 2.1.4.1) catalyzes the formation of l-homoarginine (hArg) and l-ornithine (Orn) from l-arginine (Arg) and l-lysine (Lys) (Scheme 1, left) [1,2]. AGAT also catalyzes the formation of guanidinoacetate (GAA) and Orn from Arg and glycine (Gly) (Scheme 1, right). GAA is further methylated on its non-guanidine group to the energy-related creatine by guanidinoacetate *N*-methyltransferase (GAMT; EC 2.1.1.2) [1,2]. These reactions are reversible. The corresponding equilibrium constants *K*_harg_ and *K*_gaa_ are calculated using the equilibrium concentrations of the participating amino acids (Scheme 1). hArg and GAA can be regarded as the main reaction products of the AGAT pathway. Rat kidney AGAT has a relatively broad substrate specificity [3]. hArg also functions as a substrate for AGAT and is converted to Arg and Lys by the reversed reaction (Scheme 1). Other guanidino compounds such as GAA and creatine can serve as substrates and/or inhibitors of AGAT activity [1]. GAMT and glycine *N*-methyltransferase (GNMT; EC 2.1.1.20) use *S*-adenosylmethionine (SAM) as the methyl-group donor. Gly is *N*-methylated to sarcosine (Sarc; *N*-methylglycine) by GNMT. Creatine and sarcosine undergo further reactions. Creatine cyclizes spontaneously to creatinine in a pH-dependent manner. Creatine amidinohydrolase (EC 3.5.3.3) catalyzes the hydrolysis of creatine to sarcosine and urea [4,5,6,7]. The reversible creatinine–creatine reaction is also catalyzed by creatininase (creatinine amidohydrolase; EC 3.5.2.10) [8], which is a member of the urease-related amidohydrolase superfamily. Creatininase is present in the gut [9,10].

Low circulating and low excretory concentrations of hArg are associated with worse cardiovascular outcomes and mortality [11,12,13,14,15,16,17], suggesting a particular importance of hArg in health and disease. Yet, the underlying mechanisms are still elusive and warrant further investigations. It is noteworthy that donation of a kidney by healthy humans results in a considerable decrease of circulating and urinary hArg and GAA concentrations, underlying the importance of the kidneys for hArg and GAA synthesis in humans [17].

Supplementation of hArg in diseases associated with hArg deficiency is currently being investigated. Through clinical trial simulations, a dosing regimen of 25 mg (0.133 mmol) hArg once daily was considered to lead to higher attainment of hArg reference concentrations [18]. In sport medicine studies, nitric oxide (NO) is of particular interest because NO is assumed to increase physical power. Like Arg, hArg is a potential substrate for NO synthase. Another possibility to increase NO production independent of NO synthase (NOS) is supplementation of inorganic nitrate (NO_3_^−^, O=N(O)O^−^) [19,20,21,22], presumably via its reduction to nitrite (NO_2_^−^, O=NO^−^). Surprisingly, inorganic nitrate was found to enhance the activity of dimethylarginine dimethylaminohydrolase (DDAH; EC 3.5.3.1) [23]. This may be of particular importance because DDAH hydrolyzes asymmetric dimethylarginine (ADMA), an endogenous inhibitor of NOS. Hydrolysis of ADMA by DDAH to l-citrulline and dimethylamine (DMA) would decrease the concentration of ADMA and the extent of the inhibition of NOS activity, thus increasing endogenous NO synthesis from Arg. This finding could be significant as the catalytic processes of both enzymes, i.e., AGAT and DDAH, are mechanistically very similar. Specifically, they exert the same catalytic triad and involve nucleophilic attack of the sulfhydryl (SH) group of certain cysteine (Cys) residues in the active sites of the enzymes on the carbon atom of the guanidine group of amino acids [1,2,24,25,26,27]. With respect to DDAH, it has been hypothesized that ligand binding may modulate the reactivity of the active-site Cys [27]. This possibility has thus far not been investigated mechanistically for nitrate as a ligand on DDAH or AGAT. Orn is a product of Arg-involving AGAT-catalyzed reactions (Scheme 1) and an inhibitor of AGAT activity [28].

**Scheme 2:**
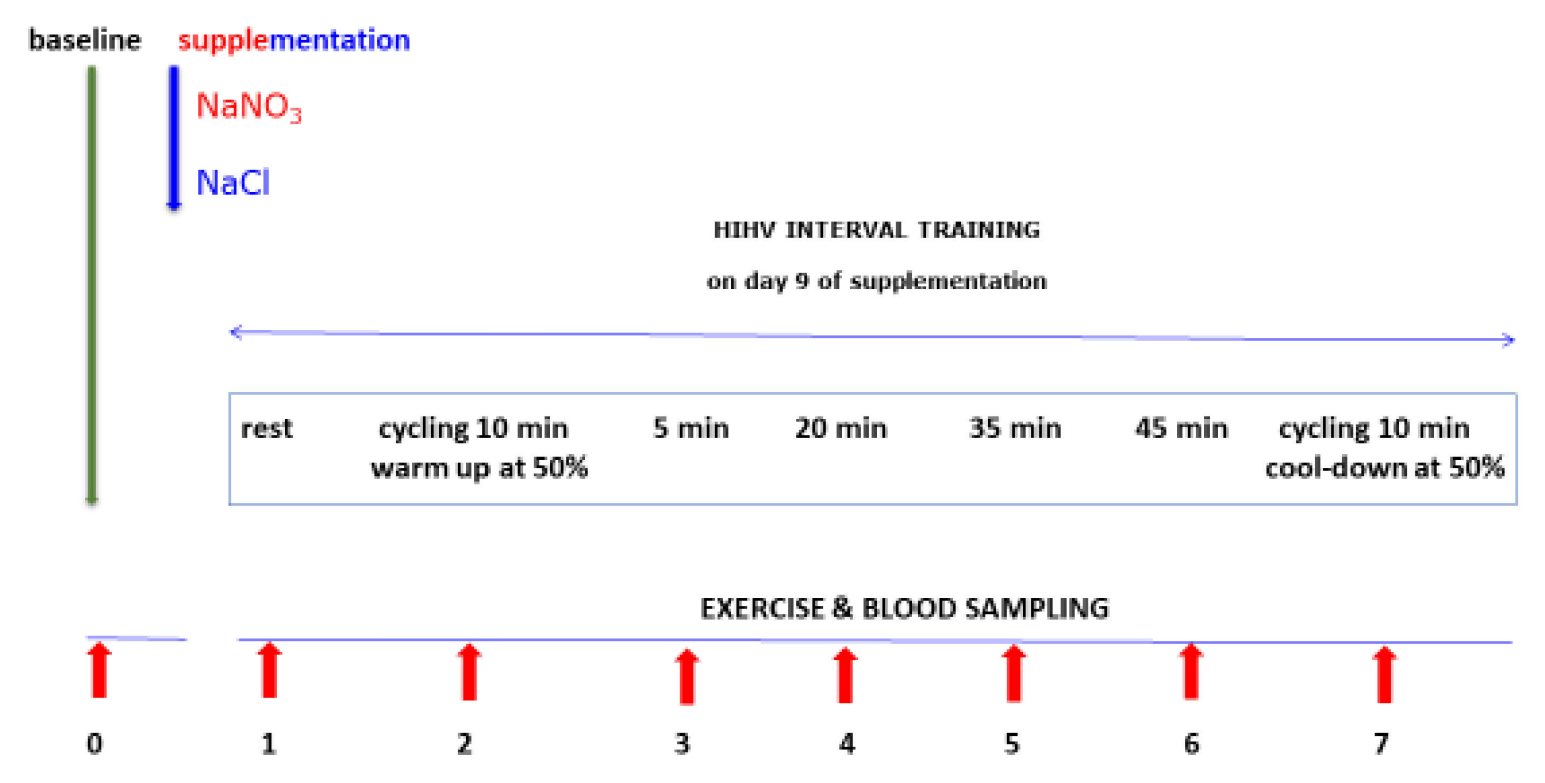
Simplified schematic of the study on the effects of supplementation of sodium nitrate (NaNO_3_) or sodium chloride (NaCl) each at a dose of 0.14 mmol/kg/day to 17 healthy young volunteers (*n* = 9 in the NaNO_3_ group, *n* = 8 in the NaCl group). Blood samples were drawn at baseline prior to supplementation (0) and at the indicated exercise timepoints (1, 2, 3, 4, 5, 6, and 7) during the high-intensity, high-volume (HIHV) interval training on day 9 of supplementation. Our group [22] previously reported the study in detail (see also Section 4).

In a previous study, we investigated the effects of supplementary sodium nitrate (NaNO_3_) in comparison to sodium chloride (NaCl), which was used as a placebo, on oxygen uptake and power on healthy young adults [22]. In that study, we collected blood plasma samples for measurement of nitrate, nitrite, and other biochemical parameters at baseline prior to supplementation and at various exercise timepoints after drug supplementation (Scheme 2).

The aim of the present study was to investigate the effects of ingested NaNO_3_ in comparison to NaCl on the amino-acid homeostasis and on oxidative stress in the volunteers of the NaNO_3_ and NaCl groups (Scheme 2). We were particularly interested in the effects of NO_3_^−^ on the two AGAT-catalyzed reactions generating hArg and GAA (Scheme 1), as well as on lipid peroxidation, which was measured as malondialdehyde (MDA) in plasma [29]. Nitrate, nitrite, amino acids, and their metabolites including hArg and GAA were measured in plasma samples by gas chromatography–mass spectrometry (GC–MS) and were used to calculate the equilibrium constants *K*_harg_ and *K*_gaa_. The results were thoroughly analyzed using different statistical methods. The main result of our study is that NaNO_3_ ingestion increases hArg synthesis independent of physical exercise. This observation has not been reported so far.

## 2. Results

### 2.1. Baseline Plasma Concentrations

At baseline, i.e., about 3 days before NaNO_3_ or NaCl supplementation started (Scheme 2), there were no statistically significant differences in the plasma concentrations of the analytes except for sarcosine (difference 40%, *p* = 0.005), indicating similar groups. The difference in sarcosine concentration is unexpected and we have no explanation for this observation. Furthermore, no statistically significant differences were found for the equilibria constants *K*_harg_ and *K*_gaa_ or their ratio *K*_harg_/*K*_gaa_ between the groups (see Supplementary Table S1). The plasma concentrations of all amino acids and their metabolites, including GAA, hArg, ADMA, and creatinine, measured in both study groups (Table S1) were closely similar to those found by us and other groups in populations of healthy subjects. There were numerous statistically significant Spearman correlations between the plasma concentrations of the measured amino acids (Table S2). The largest number of correlations was observed for Thr (*n* = 14) and Gly (*n* = 12). The strongest correlation coefficient was observed between Asp/Asn and Phe (*r* = 0.882, *p* = 8 × 10^−6^).

### 2.2. Effect of Nitrate Supplementation on Plasma Nitrate and Nitrite Concentrations

Expectedly, supplementation of NaNO_3_ resulted in statistically significantly higher plasma nitrate and nitrite concentrations than supplementation of NaCl (Table 1). Median plasma nitrate and nitrite concentrations were 7.4-fold and 1.7-fold higher in the NaNO_3_ group compared to the NaCl group, respectively. These results indicate that an apparently very small fraction of about 0.26% of ingested NaNO_3_ is converted to nitrite, presumably due to nitrate reductase activity of the mouth and gut flora of the subjects who ingested NaNO_3_ [19,20]. The area under the curve (AUC) value of the receiver operating characteristic curves (ROC), i.e., the AUC-ROC value, of plasma nitrite was higher than that of nitrate and of all amino acids and malondialdehyde (MDA) measured in the study (Table 1; Figure S1). Thus, plasma nitrite turned out to be a more discriminant marker of NaNO_3_ ingestion than plasma nitrate.

### 2.3. Effect of Supplementation on Plasma Amino Acids Concentrations

**Figure 1:**
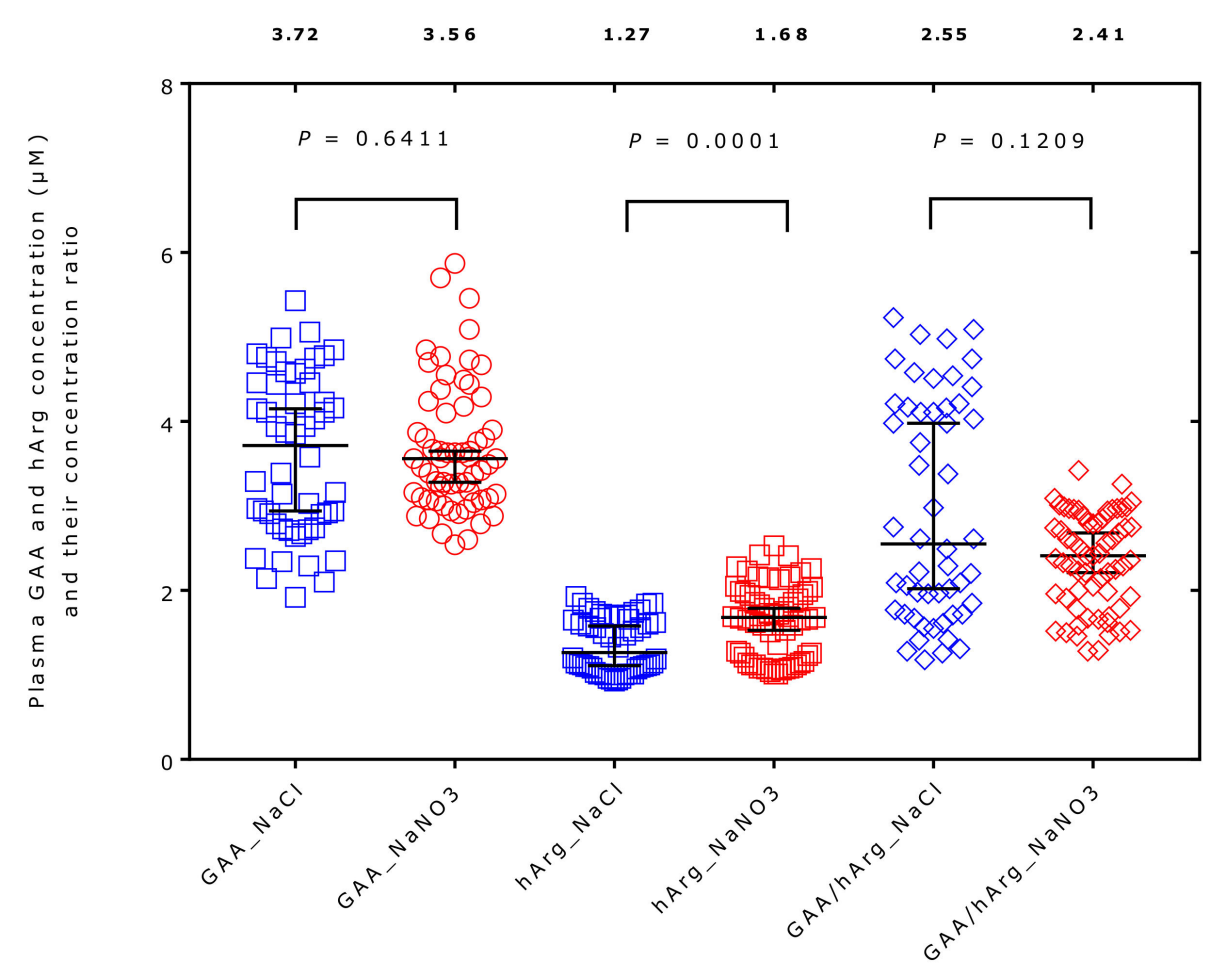
Plasma concentrations of guanidinoacetate (GAA) and homoarginine (hArg) and their molar ratio (GAA/hArg) in the NaCl and NaNO_3_ groups after supplementation of NaCl (*n* = 52, 54) or NaNO_3_ (*n* = 63). The plasma concentrations measured at all exercise timepoints were considered. Data are presented as the median with 95% confidence interval. Numbers on the top are median values. Mann–Whitney test was performed between the groups for GAA, hArg, and GAA/hArg.

Compared to NaCl, NaNO_3_ supplementation resulted partly in lower and partly in higher plasma concentrations of certain amino acids (Table 1). The greatest and statistically significant difference was obtained for hArg (+24%) in the NaNO_3_ group (Figure 1). The plasma concentrations of Orn/Cit (+16%) and Arg (+14%) were also higher in the NaNO_3_ group, yet they did not result in a significant change of the global arginine bioavailability ratio (GABR = [Arg]/[Orn/Cit]), because they increased similarly.

### 2.4. Effect of Supplementation on the Equilibrium Constants of the AGAT-Catalyzed Reactions Producing Guanidinoacetate and Homoarginine

**Figure 2:**
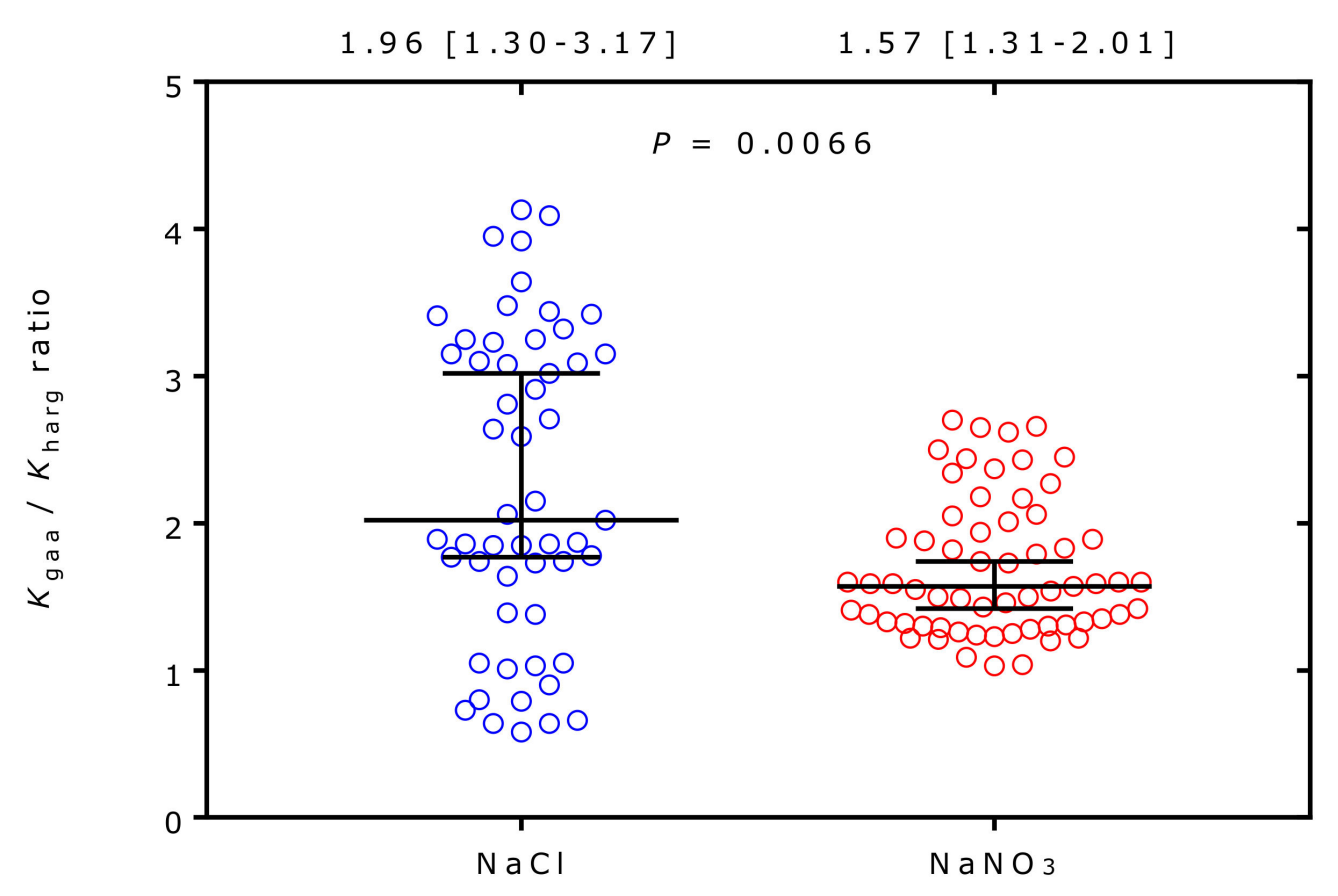
*K*_gaa_/*K*_harg_ ratios in the NaCl group (*n* = 53) compared to the NaNO_3_ group (*n* = 63) on day 9 after supplementation. The values determined at all exercise timepoints in each group were considered and presented as the median with 95% confidence interval.

We calculated the equilibrium constants *K*_harg_ and *K*_gaa_ of the two major AGAT-catalyzed reactions in the two study groups after supplementation (Table 1). The median *K*_harg_ value was 0.0035 in the NaCl group and 0.0049 in the NaNO_3_ group (*p* < 0.0001). The median *K*_gaa_ value was 0.00685 in the NaCl group and 0.0077 in the NaNO_3_ group (*p* = 0.0334). The median *K*_gaa_/*K*_harg_ ratio was 1.96 in the NaCl group and 1.57 in the NaNO_3_ group (*p* = 0.0066) (Figure 2). These results indicate that inorganic nitrate supplementation is associated with a higher hArg formation compared to the GAA formation. The *K*_gaa_, *K*_harg_, and *K*_gaa_/*K*_harg_ ratio values in the plasma samples of the present study are comparable with those at baseline and those measured in several rat organs [30].

### 2.5. Effect of Supplementation and Exercise on the Equilibrium Constants in Each Volunteer and at Each Exercise Timepoint

**Figure 3:**
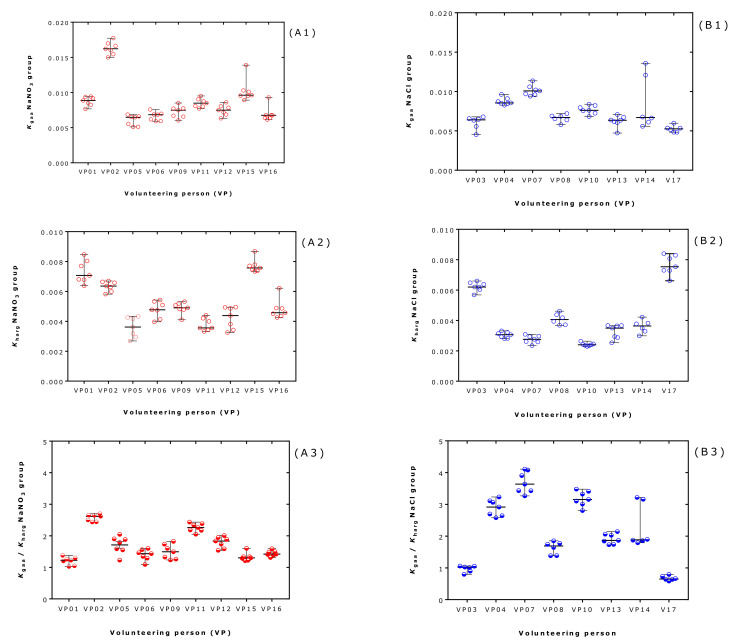
*K*_gaa_, *K*_harg_, and *K*_gaa_/*K*_harg_ values of the volunteering persons (VP) of the NaNO_3_ group (**A1**–**A3**) and of the NaCl group (**B1**–**B3**) at the seven timepoints of exercise after supplementation (see Scheme 2). Data are shown as the median with 95% confidence interval for each volunteer (see also Figure 4).

**Figure 4:**
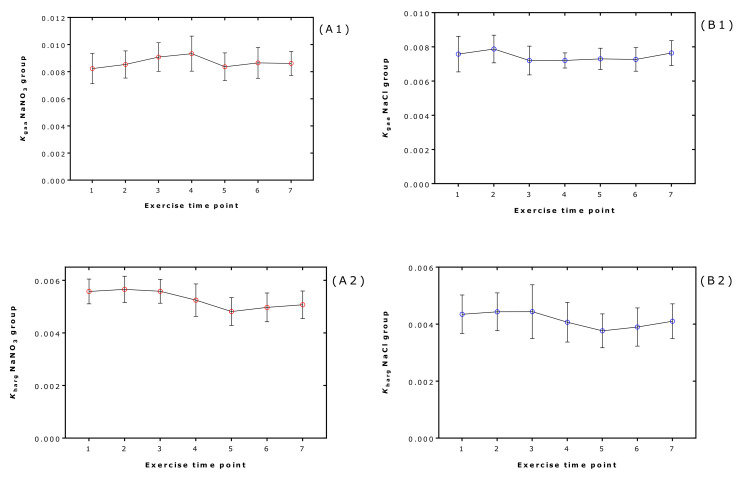
*K*_gaa_, *K*_harg_, and *K*_gaa_/*K*_harg_ values of the volunteers of the NaNO_3_ group (**A1**,**A2**) and of the NaCl group (**B1**,**B2**) at the seven individual timepoints of exercise after supplementation (see Scheme 2). The number of the samples was too small for the normality test; the data are shown as mean with standard error of the mean for the volunteers of the NaNO_3_ and NaCl groups (see also Figure 3).

Figure 3 shows the *K*_gaa_, *K*_harg_, and *K*_gaa_/*K*_harg_ ratio values for each volunteer of both study groups at all exercise timepoints. Figure 4 shows the mean values of *K*_gaa_, *K*_harg_, and *K*_gaa_/*K*_harg_ of the volunteers in the NaNO_3_ and NaCl groups at each exercise timepoint. The data of Figure 3 and Figure 4 indicate that the parameters *K*_gaa_, *K*_harg_, and *K*_gaa_/*K*_harg_ ratio varied within and between the volunteers during the exercises.

**Figure 5:**
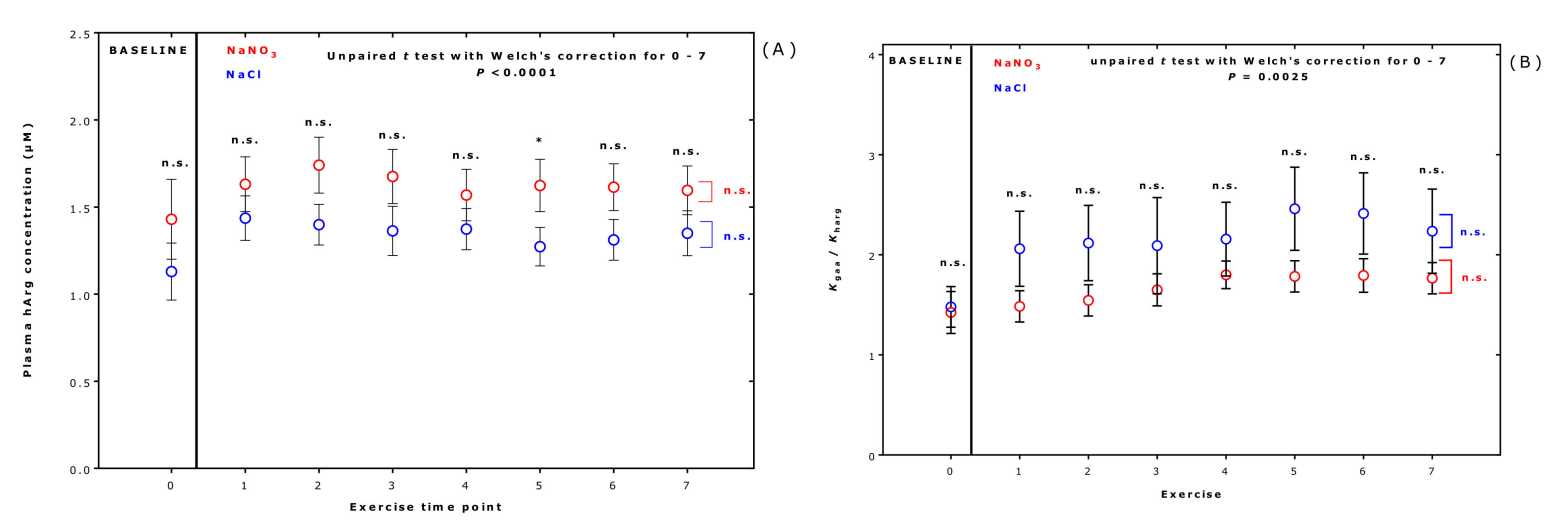
Plasma homoarginine (hArg) concentration (**A**) and *K*_gaa_/*K*_harg_ (**B**) values of the volunteering persons of the NaNO_3_ and NaCl groups at baseline (0) and at the seven individual timepoints of exercise after supplementation (see Scheme 2). Data are shown as the mean with standard error of the mean for the volunteers of the NaNO_3_ and NaCl groups. Unpaired *t*-test with Welch’s correction was performed. n.s., not significant.

Figure 5 shows the plasma hArg concentrations and the *K*_gaa_/*K*_harg_ ratio values for each volunteer of both study groups at baseline and at each exercise timepoint for the volunteers of the NaNO_3_ and NaCl groups. At baseline, neither the plasma hArg concentrations (1.43 ± 0.23 vs. 1.13 ± 0.16 µM) (Figure 5A) nor the *K*_gaa_/*K*_harg_ ratio values differed between the groups (1.48 ± 0.204 vs. 1.42 ± 0.210) (Figure 5B). During exercise, the plasma hArg concentration did not change except at exercise timepoint 5 (1.62 ± 0.15 vs. 1.27 ± 0.11 µM). The *K*_gaa_/*K*_harg_ ratio also did not change during exercise. Differences (unpaired *t*-test with Welch’s correction) between the groups were observed for the plasma hArg concentration (1.61 ± 0.03 vs. 1.33 ± 0.03 µM; *p* < 0.0001) and the *K*_gaa_/*K*_harg_ ratio (2.22 ± 0.26 vs. 1.66 ± 0.13; *p* = 0.0025) when considering the data at baseline and at exercise.

### 2.6. Effects of Supplementation and Exercise on Plasma Creatinine in Each Volunteer and at Each Exercise Timepoint

At baseline, i.e., prior to supplementation, the plasma concentration of creatinine did not differ between the groups: 92 [84–101] µM in the NaCl group vs. 87.5 [81.6–96.5] µM in the NaNO_3_ group (*p* = 0.497; Table S1). When considering all volunteers and all exercise timepoints, the plasma creatinine concentrations did not differ between the NaCl and NaNO_3_ groups (129.4 ± 23.4 µM vs. 126.1 ± 23.7 µM; *p* = 0.4402, unpaired *t*-test; Table 1). Figure S2 shows that the plasma creatinine concentration increased over the whole exercise range after supplementation with NaCl or NaNO_3_. The difference between the groups was statistically significant (*p* = 0.0083, ANOVA). Under the consideration that all exercise points (*x*) are equidistant, the plasma creatinine concentration (*y*, µM) increased linearly: *y* = 104 + 5.6 *x*, *r*^2^ = 0.9782, for the NaCl group; *y* = 109 + 5.2 *x*, *r*^2^ = 0.9443, for the NaNO_3_ group. Thus, the increases in plasma creatinine concentration were due to exercise rather than the result of the supplementation of NaCl or NaNO_3_. There was no difference when comparing the respective exercise time points.

### 2.7. Effects of Supplementation and Exercise on Plasma Malondialdehyde and Potassium Ions

The plasma MDA concentrations did not change after supplementation of NaNO_3_ or NaCl in the volunteers over the whole exercise time range (Figure S3). When considering all volunteers and exercise timepoints, the plasma MDA concentrations differed between the groups (*p* = 0.0002, Mann–Whitney test; Table 1).

Plasma potassium levels at baseline were similar (4.15 ± 0.41 mM in the NaCl group and 3.92 ± 0.21 mM in NaNO_3_ group; *p* = 0.1536) (Table S1). After supplementation at exercise timepoint 1, the concentration of the K^+^ concentration in the plasma samples was 4.14 ± 0.21 mM in the subjects of the NaCl group and 3.96 ± 0.24 mM in the subjects of the NaNO_3_ group, and they did not differ statistically (*p* = 0.1252). After supplementation at exercise timepoint 2, the concentration of the potassium ions in the plasma samples was 4.54 ± 0.25 mM in the subjects of the NaCl group and 4.45 ± 0.27 mM in the subjects of the NaNO_3_ group, and they did not differ statistically (*p* = 0.5289). Within each group, the plasma K^+^ concentration was statistically significantly higher at exercise timepoint 2 compared to exercise timepoint 1 (Figure S4). The plasma K^+^ concentration increased very similarly and in parallel in the subjects of both groups, and the difference persisted over the whole exercise period (Figure S5).

**Figure 6:**
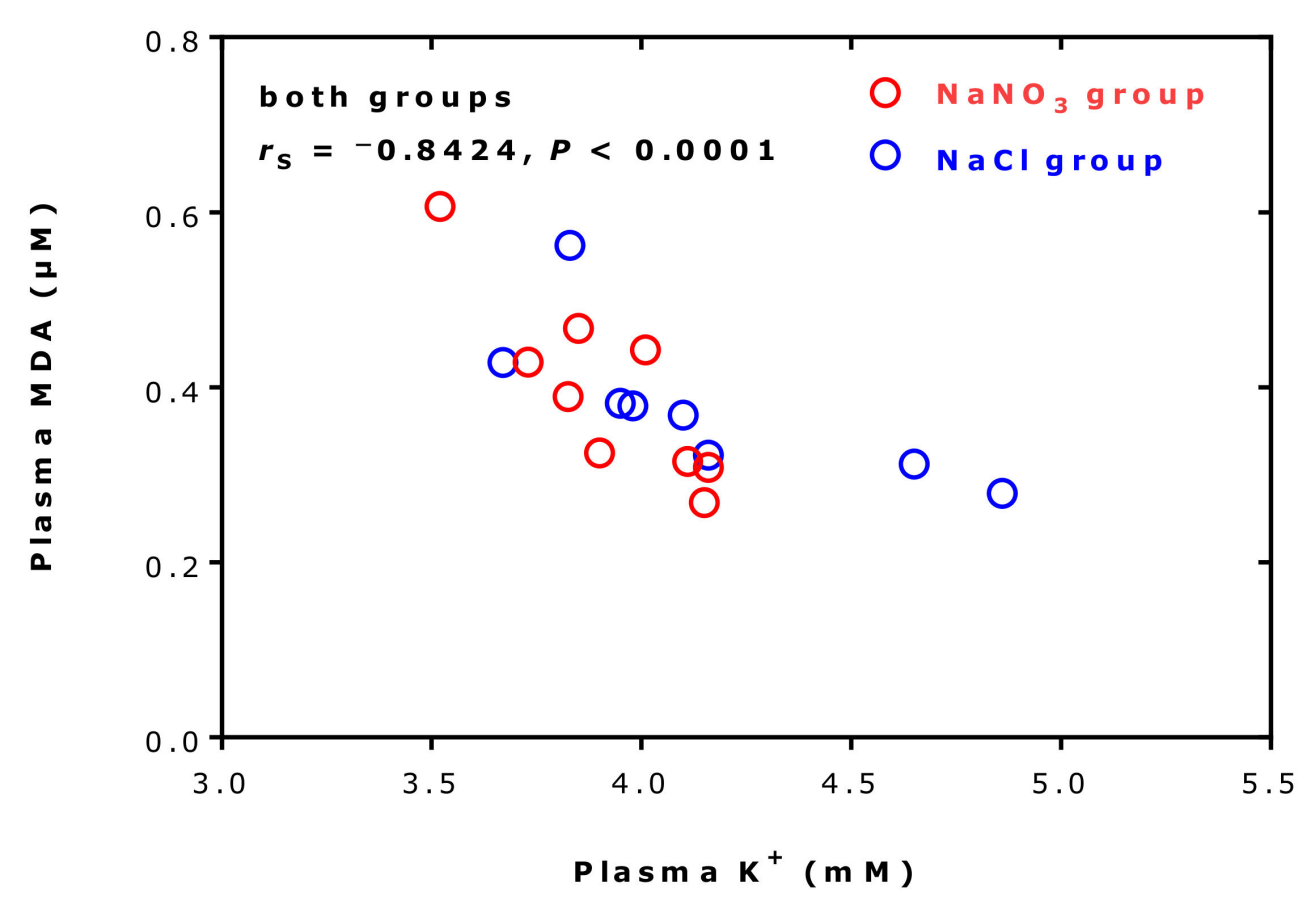
Relationship between the baseline plasma MDA and K^+^ concentrations in the volunteering persons of the NaCl and NaNO_3_ groups. Each symbol corresponds to a single volunteering person.

At baseline, prior to supplementation with NaCl or NaNO_3_, the plasma concentrations of MDA and K^+^ correlated inversely with each other (Figure 6). The Spearman correlation coefficient *r*_S_ was −0.8424 (*p* < 0.0002) in the combined groups. The Pearson correlation coefficient *r*_P_ was −0.8831 (*p* = 0.0027) in the NaNO_3_ group and −0.7505 (*p* = 0.0319) in the NaCl group. No correlation was found between plasma MDA and sodium ion (Na^+^) concentration (*r*_S_ = −0.2426, *p* = 0.3467) of the combined groups. Considering all data after supplementation, no correlation was found between the plasma MDA and K^+^ concentrations in the NaNO_3_ group (*r*_S_ = 0.1684, *p* = 0.187), whereas the correlation was positive in the NaCl group (*r*_S_ = 0.3467, *p* = 0.0089). A positive correlation between plasma MDA and K^+^ concentrations was observed when considering the combined groups and all exercise timepoints (*r*_S_ = 0.2728, *p* = 0.0027). At the exercise timepoint 7, a positive correlation was found between plasma MDA and K^+^ concentrations in the NaCl group (*r*_P_ = 0.8423, *p* = 0.0087).

**Figure 7:**
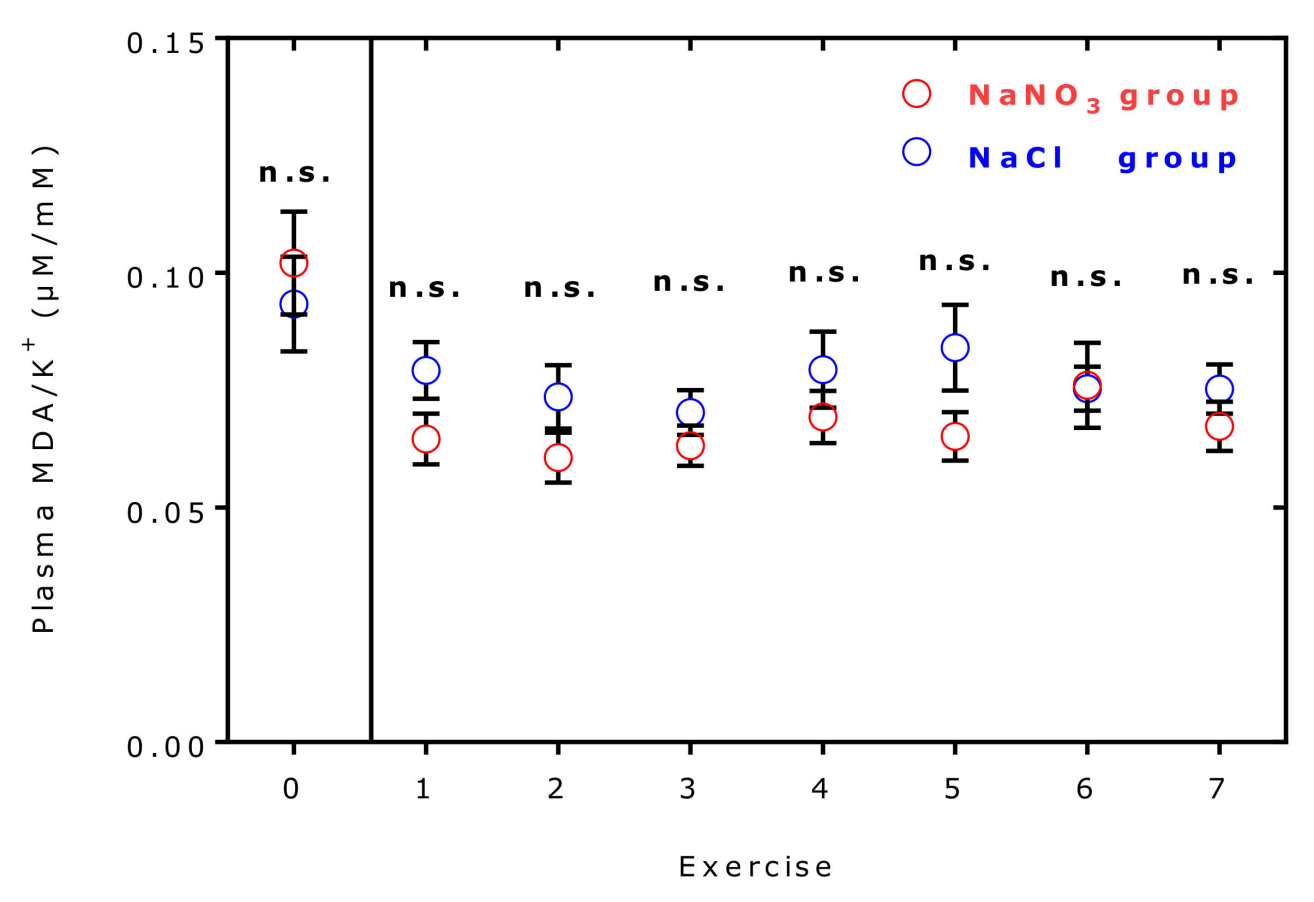
Molar ratio of plasma malondialdehyde (MDA) to plasma potassium (K^+^) in the volunteering persons of the NaNO_3_ and NaCl groups at baseline and at the seven exercise timepoints after supplementation of NaCl or NaNO_3_ (see Scheme 2). Data are shown as the mean with standard error of the mean. n.s., not significant (unpaired *t* test with Welch’s correction).

At baseline, the plasma MDA/K^+^ did not differ between the NaNO_3_ and NaCl groups (0.102 ± 0.033 vs. 0.093 ± 0.029 µM/mM; *p* = 0.5668; unpaired *t*-test with Welch’s correction). Figure 7 shows that the molar ratio of plasma MDA to plasma K^+^ in the volunteering persons of the NaNO_3_ and NaCl groups changed almost in parallel at the seven exercise timepoints.

### 2.8. Multivariate Analyses

**Figure 8:**
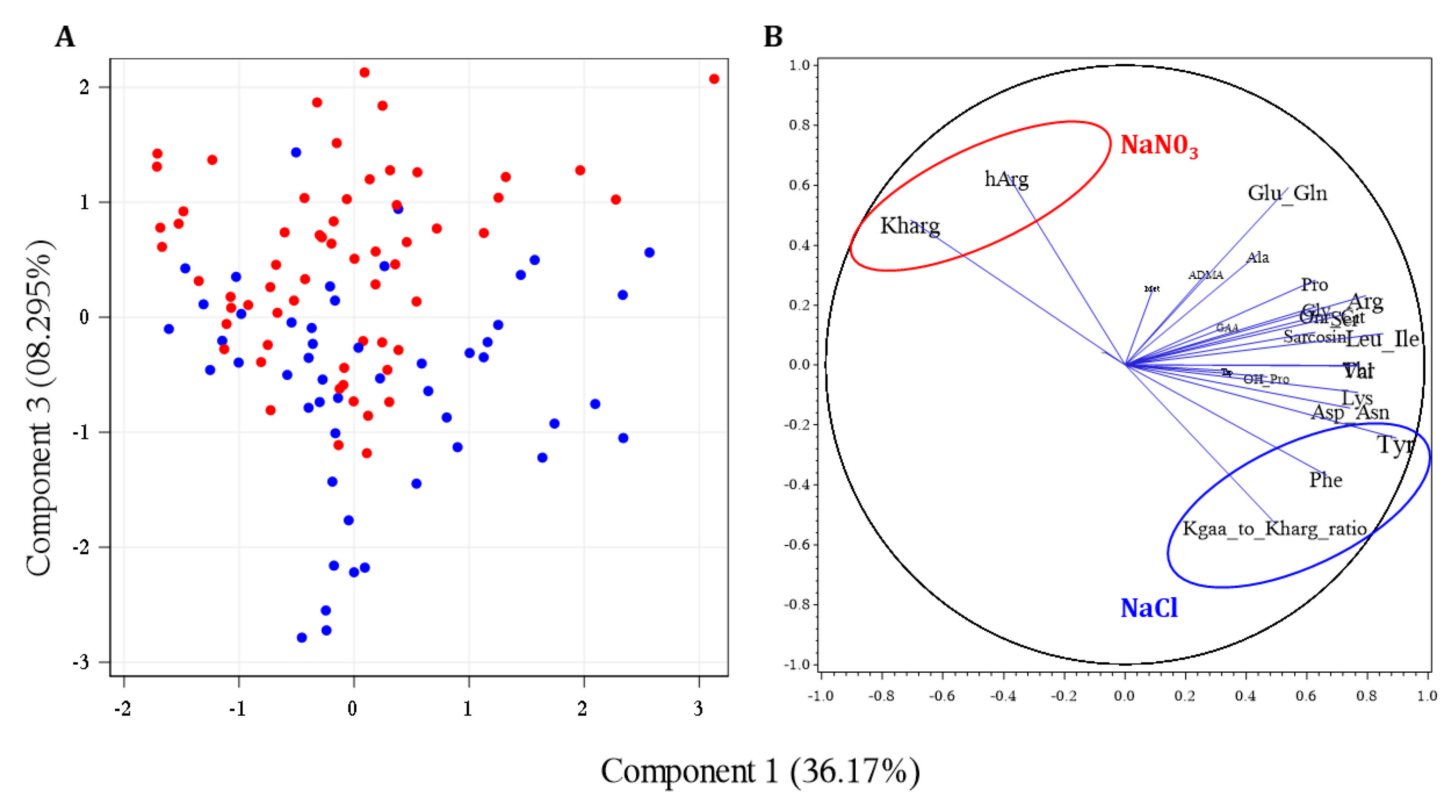
Score plot of observations (**A**) and variables factor map (**B**) projected onto principal component 1 and principal component 3.

Multivariate statistical analyses were used to describe the relationships among plasma amino-acid concentrations and related parameters such as *K*_gaa_, *K*_harg_, time, and kind of treatment (NaNO_3_ vs. NaCl). Because plasma amino-acid concentrations are largely correlated each other (see Figure S6), we used both supervised and unsupervised a posteriori approaches to reduce the data and identify underlying constructs or patterns that best explain data according to supplementation. In the principal component analysis, we used 25 variables, including amino acids, equilibrium constants, and time. These analyses are described in detail in the Supplementary Materials (Figure S7, Figure S8, Table S3, Table S4). We found that the C1/C3 factorial plane best discriminated the effect of NaNO_3_ supplementation compared to NaCl supplementation. Five variables are well represented in this figure, indicating that NaNO_3_ supplementation results in higher plasma hArg and a higher *K*_harg_ equilibrium constant, while NaCl supplementation results in higher plasma aromatic amino acids and in a higher *K*_gaa_/K_harg_ ratio (Figure 8). Thus, multivariate analyses strongly suggest that the effect of supplemented NaNO_3_ as compared to supplemented NaCl mainly relates to changes in plasma hArg.

## 3. Discussion

Nitric oxide (NO) is endogenously produced from l-arginine by the catalytical action of NO synthases [31]. NO is a signaling molecule with multiple physiological actions such as vasodilatation and inhibition of platelet aggregation [31]. NO is oxidized by oxyhemoglobin to inorganic nitrate (NO_3_^−^, O=N(–O)O^−^). Inorganic nitrite (NO_2_^−^, O=N–O^−^) is the autoxidation product of NO. In healthy men, we found a positive correlation between plasma nitrite from endogenous sources and performance during high-intensity exercise, but not between performance and oxidative stress [32]. Nitrite and nitrate are interconvertible: nitrite can be oxidized by oxyhemoglobin to nitrate, whereas nitrate can be reduced to nitrite by bacterial nitrate reductases, which are abundantly present in the mouth and gut flora. Under certain conditions, nitrite can be converted to NO via chemical and enzymatic reactions [31]. The nitrate/nitrite/nitric oxide cycle is considered a recyclable contributor to endogenous NO derived from the l-arginine/NO synthase pathway. Salts of inorganic nitrate rather than of nitrite are experimentally used in animal and human studies mainly to support endogenous production of NO in cases of NO deficiency. In addition to diseases associated with NO deficiency, sport in medicine is currently the main experimental research area of nitrate supplementation aimed at enhancing physical performance [19,20,21,22]. Yet, thus far, the outcome of such studies is contradictory [21], and the well-known health risks, notably the cancerogenic potential of nitrite, are currently disregarded in nitrate supplementation studies.

In a previous study, we investigated the effects of supplementary NaNO_3_ in comparison to NaCl as a placebo on the relationship between O_2_ uptake and power at different intensities [22]. The results of that study indicated that nitrate supplementation has a long-term effect for at least 7 days after cessation during heavy all-out workloads yet without affecting endurance capacity [22]. In the present study, we investigated the effects of supplementary NaNO_3_ and NaCl on the homeostasis of amino acids and oxidative stress in the plasma samples collected previously [22]. The focus of our present study was on the possible effects of nitrate supplementation on hArg and GAA and biochemically related amino acids involved in the pathways, where hArg and GAA are biosynthesized by AGAT (Scheme 1). Physical exercise is often considered to be associated with oxidative stress [33,34,35]. We, therefore, measured in our study plasma MDA, a generally accepted and commonly measured biomarker of oxidative stress in clinical and sport medicine studies, specifically of lipid peroxidation [29].

We found that daily supplementation of 0.1 mmol NaNO_3_/kg body weight for 9 days to healthy young men resulted in sevenfold higher plasma concentrations of nitrate compared to healthy young men who ingested daily 0.1 mmol NaCl/kg body weight. With respect to plasma nitrite, we found 1.7-fold higher plasma nitrite concentrations in the NaNO_3_ group compared to the NaCl group. The highest ROC-AUC values were obtained for plasma nitrite concentration, indicating a high predictivity of nitrite for nitrate ingestion than plasma nitrate or any other of the investigated amino acids.

Assuming an adult subject with a body weight of 80 kg, the dose of 0.1 mmol NaNO_3_/kg body weight per day would correspond to a supplemented nitrate amount of 8 mmol. This amount is about seven times higher than the NO amount daily produced by healthy young volunteers [31,36]. Recently, the modified Delphi technique was used to establish the views of 12 expert panel members on the use of dietary nitrate as an ergogenic aid [37]. “It is recommended that athletes looking to benefit from dietary nitrate supplementation should consume 8–16 mmol nitrate acutely or 4–16 mmol/day nitrate chronically (with the final dose ingested 2–4 h pre-exercise) to maximize ergogenic effects, taking into consideration that, from a safety perspective, athletes may be best advised to increase their intake of nitrate via vegetables and vegetable juices. Acute nitrate supplementation up to ~16 mmol is believed to be safe, although the safety of chronic nitrate supplementation requires further investigation.” [37]. The NaNO_3_ dose used in our study (here and in [22]) is close to the lowest recommended dose of inorganic nitrate [37].

Compared to NaCl ingestion, NaNO_3_ supplementation influenced differently the homeostasis of plasma amino acids. Of particular interest is the remarkable higher hArg plasma concentrations in the subjects who ingested NaNO_3_ compared to those who ingested NaCl (placebo). Apparently, NaNO_3_ shifted the equilibria of the two reactions catalyzed by AGAT, i.e., the biosynthesis of hArg and GAA, in favor of hArg. This is of particular importance because low hArg synthesis is associated with higher morbidity in the renal and cardiovascular systems [11,12,13,14,15,16,17]. The higher plasma hArg concentrations on the order of 28% in the NaNO_3_ group are comparable to the average decrease in plasma and urine hArg concentrations observed in healthy subjects who donated a kidney [17].

The mechanisms via which ingested inorganic nitrate increased plasma hArg concentrations in the present study are unknown. Similar nitrate effects were observed for the DDAH activity [23]. The activity of AGAT and DDAH is exerted by certain Cys moieties in the active centers of AGAT and DDAH. One may hypothesize that inorganic nitrate predominantly increases the AGAT-catalyzed formation of hArg. Yet, the underlying mechanisms are still incompletely understood. Inorganic nitrate is the base of the very strong nitric acid (p*K*_a_, −1.37). Previous mechanistic studies with isolated AGAT proposed that some ligands bind to AGAT and facilitate the deprotonation of the SH group of the Cys residue of AGAT that attacks the guanidino group of AGAT substrates, notably of Arg and hArg [27]. Such a mechanism could have occurred in our study. The few mM concentrations of nitrate in plasma reached after nitrate supplementation suggest that similar concentrations could have been reached within AGAT-expressing cells. In such a case, nitrate binding to AGAT should facilitate more strongly the formation of hArg rather than of GAA. Inorganic nitrite is the base of the weak nitrous acid (p*K*_a_, 3.29). In theory, nitrite could be involved in the AGAT activity by *S*-nitrosylating the Cys group in active center of AGAT, analogous to DDAH [25]. Yet, the relatively low nitrite concentrations could be too low to act like nitrate by facilitating the deprotonation of Cys or through its *S*-nitrosylation. To our knowledge, such mechanistic studies have not been performed so far with AGAT or DDAH. As nitrate ingestion can alter the oral and gut microbiome [38,39,40,41], we cannot exclude that this also occurred in our study. It is notable that hArg is a potent inhibitor (IC_50_, 0.16 µM) of bacterial growth including *Escherichia coli B* [42,43]. hArg synthesis from Lys has been reported for bacterial cytochrome *c* [44].

In addition, given the high concentrations of nitrate resulting from ingestion of inorganic nitrate, interaction of nitrate with renal Arg and ADMA transporters [45,46] could also have contributed to our results, for instance, by binding nitrate (an anion) on two arginine residues (cations) in a high-affinity nitrate transporter [46].

Supplementation of 15 g (85.6 mmol) l-citrulline per day to humans was found to increase the serum concentration of hArg and GAA to a closely comparable extent, most likely by increasing the bioavailability of l-arginine from ingested l-citrulline [47]. We are not aware whether ingestion of nitrate increases the bioavailability of Arg from Cit. In our study, we did not observe a change in GABR upon NaNO_3_ compared to NaCl. The different effects of ingested NaNO_3_ (present study) and l-citrulline [47] on hArg and GAA synthesis suggests that nitrate influences AGAT activity by mechanisms other than those increasing the bioavailability of Arg and Lys. A possibility could be increasing AGAT activity by facilitating the deprotonation of the key Cys residue in the active site of the enzyme.

These considerations suggest that chronic supplementation of 0.133 mmol hArg per day might be more effective than supplementation of 7 mmol NaNO_3_ or 86 mmol l-citrulline in increasing circulating hArg concentrations in humans to concentration ranges that are considered normal [18]. Our results indicate that an apparently very small fraction of about 0.26% of ingested NaNO_3_ is converted to nitrite. One important reason might be the 10-fold larger NaNO_3_ excretion during the exercise period in the NaNO_3_ group compared to placebo (Lützow et al., unpublished). This order of magnitude was also reached for serum nitrite after oral supplementation of about 0.4 mmol ISDN/day or 0.8 mmol PETN/day for 5 days each [48]. However, it is unclear whether nitrite derived from inorganic nitrate or organic nitrates at doses used thus far are able to exert pharmacological effects and/or enhance physical power.

Prior to supplementation, we observed a strong negative correlation between the concentrations of plasma MDA and K^+^ in the resting volunteers of both study groups. After supplementation, this correlation disappeared in both groups, suggesting that this is not an effect of nitrate or chloride supplementation. A possible explanation for this and the increase in plasma K^+^ and MDA could be hemolysis, which is a common and non-negligible phenomenon during blood laboratory sampling worldwide and concerns K^+^ and MDA, among many other biochemical parameters [49,50,51,52]. This especially applies to analytes such as K^+^, which are abundantly present in human erythrocytes (about 80–100 mM K^+^) and at relatively low (3–5 mM K^+^) concentrations in blood plasma [49,50,51,52,53]. We found that plasma MDA may also be associated with hemolysis in healthy humans [54]. Cyclooxygenase 1 (COX-1) in human platelets is a major source for MDA [29] and can be inhibited by acetylsalicylic acid (aspirin) [55]. The volunteers whoparticipated in our study did not take any drugs; hence, the increase in plasma MDA concentration seen in our study is likely to be due to hemolysis. In our study, plasma K^+^ concentration increased on average by 0.48 mM in the NaCl group and by 0.58 mM in the NaNO_3_ group, suggesting no appreciable effect of ingested nitrate on plasma K^+^ concentration and presumably no effects on hemolysis and oxidative stress. We assume that some plasma K^+^ originated from exercise-induced muscle damage [33]. Previously, we found a positive correlation of endogenous plasma nitrite concentration with physical performance during high-intensity exercise but not with oxidative stress [32]. For completeness, it should be mentioned that other systems such as Na^+^/K^+^-ATPase in human erythrocytes and K^+^ loss from the working muscles due to the electrical activity during exercise [56] may also play a role in physical exercise and oxidative stress, as reported in prehypertensive patients [57]. Additional potential mechanisms could be elevation of oxidative stress by K^+^-induced depolarization [58] and NADPH oxidase [59].

Physical exercise and oxidative stress have been discussed for several decades to be closely interrelated [60]. Yet, many issues in this area, including the supplementation of antioxidants and, more recently, the ingestion of inorganic nitrate, remain inconclusive [61,62,63,64]. In a recent study, acute supplementation of nitrate for 4 h compared to chloride did not affect brachial and femoral flow-mediated dilatation, and did not change blood pressure or heart rate in abdominally obese men [65]. This trial did not provide evidence for effects of a single dose of inorganic nitrate on 4 h vascular endothelial function.

Previous and present studies from our groups on supplementation of inorganic nitrate in the low, recommended dose as an ergogenic aid to young healthy subjects, suggest that the benefit is rather small. Nevertheless, inorganic nitrate supplementation has a long-term effect for at least 7 days after cessation during heavy all-out workloads, albeit with no appreciable endurance capacity [22].

Our study showed that short-term NaNO_3_ supplementation alters whole-body amino-acid homeostasis compared to NaCl, but seems not to affect oxidative stress. Hemolysis is inevitable, and even low-extent hemolysis may hamper the significance of biomarkers of oxidative stress such as MDA and other thiobarbituric acid-reactive substances (TBARS), highlighting the role of oxidative stress in physiology and sports. A potential alternative could be the measurement of MDA in urine samples in sport medicine studies [66]. In the case of measuring MDA in plasma, plasma K^+^ could be tested as a possibility to correct for hemolysis, as plasma K^+^ concentration increases proportionally with the hemolysis degree [51]. As the concentration of circulating MDA is dependent upon study design even in placebo-controlled studies and the type of the anticoagulation, special care must be taken when using very similar blood sampling and storage conditions including storage time [67]. Oxidative stress can be measured in humans by a large number of oxidative stress biomarkers including glutathione in reduced and oxidized forms, superoxide dismutase, and catalase activity [68].

## 4. Materials and Methods

### 4.1. Study Design and Subjects

This work is part of a training study that was designed as a double-blind, randomized controlled trial [22]. The Ethics Committee of the Hannover Medical School approved the study protocol (code number 2015–2013). The volunteers were all athletes and recruited by the Institute of Sport Medicine and the Institute of Sport Science. Seventeen healthy male subjects (age, 26.7 ± 4.2 years; range, 19 to 35 years; body height, 182.7 ± 7.4 cm; body mass: 77.9 ± 9.8 kg) gave written informed consent to participate in this study. The volunteers of the placebo (NaCl) and verum (NaNO_3_) groups did not differ with respect to age (26.5 ± 3.4 vs. 26.1 ± 4.4 years; *p* = 0.85) or body weight (77.2 ± 10.9 vs. 78.3 ± 8.0 kg; *p* = 0.84) None of the participants smoked or took any medication. To generate two equally balanced groups, the subjects were assigned by body weight, relative peak power, and relative VO_2max_ (maximal oxygen consumption) measured on the equipment Metalizer 3B (Cortex, Germany), which were recorded during the initial incremental test (IT). After the assignment, the data revealed no significant differences in absolute and relative mean power between the groups. The test persons were assigned to the verum NaNO_3_ group (N) and the placebo NaCl group (PL). Then, they followed the same high-intensity, high-volume (HIHVT) training protocol. One training session was performed on day 9 of the supplementation (Scheme 2).

### 4.2. Sodium Nitrate and Sodium Chloride Supplementation

The healthy young volunteers receive the same daily dose of 0.14 mmol/kg NaNO_3_ or NaCl. The daily nitrate supplementation was taken by nine subjects in the form of NaNO_3_ at a dose of 8.5 mg per kg body weight dissolved in 250 to 500 mL of tap water (NaNO_3_ group; 4.85 ± 0.62 W/kg). NaCl served as a placebo dissolved in 250 mL of water and was taken by eight subjects at the same dose (NaCl group; 4.83 ± 0.91 W/kg). Both supplements were consumed in portions throughout the day. The volunteers did not fast overnight. Volunteers were instructed to avoid nitrate-rich food such as beetroot and not to change their usual diet. There were no other restrictions, such as the use of oral mouthwashes or caffeine consumption, in order to not interfere with lifestyle behavior or training conditions. The beverages were rationed individually over the whole day.

Solid NaNO_3_ and NaCl were from Merck KGaA (Darmstadt, Germany). The two beverages were not distinguishable by their taste. The sodium salts of the supplements were considered to exclude the possible effects of ingested sodium ions in the study. After eight days of supplementation, the exemplary training session was performed.

### 4.3. Physical Exercise and Blood Sampling

All physical tests were carried out on a cycle ergometer (Excalibur Sport, Lode, Netherlands). Each test was carried out in the same daytime for the same subject. The individual height of the saddle and handlebar was adjusted in each test and training session.

In the first week of the trial, pretests were performed. To determine the maximum power of each participant, an incremental test was accomplished. The data from this test were the basis for the training. These tests were performed before any supplementation was given.

During the second to fourth week, the participants had to exercise three times a week, in addition to their individual recreational workouts. The subjects were instructed not to make any changes in their regular workout routine during the trial. One exercise session consisted of 45 intervals of each 30 s HIHVT workout with maximum power (determined during the incremental test) minus 10 W and 30 s of active recovery at 10 W [22]. On day 9 of the supplementation, an exercise session was performed to investigate the acute physical and biochemical effects of the supplementation. Subjects cycled for 2 min at 10 W and then for 10 min at 50% of their maximum power to warm up, then performed the 45 intervals of high intensity, and finally cycled again for 10 min at 50% W_max_. Measurement points were at rest (Rest), at the end of the warmup (A10′50%), after the intervals of 5 min (5′IT), 20 min (20′IT), 35 min (35′IT), 45 min (45′IT), and at the end of the cool-down phase (N10′50%) (Scheme 2). For the sake of simplicity, these seven exercise timepoints at which blood was drawn for biochemical analyses are abbreviated as 1, 2, 3, 4, 5, 6, and 7, respectively (Scheme 2).

Blood samples were taken at every measuring point during the trial (Scheme 2). For that, a catheter was brought in the cubital vein of one forearm and kept patent by injections of physiological saline (NaCl 0.9%, B. Braun, Melsungen, Germany) after each sample. The blood sample consisted of 8 mL of venous (v) blood taken into EDTA-blood collection tubes (Monovetten^®^, Sarstedt, Nürnberg, Germany). A part of the blood sample was immediately used to measure electrolytes ([Na^+^]_v_, [K^+^]_v_, [Cl^−^]_v_), the acid–base status, [HbO_2_] (ABL 505, Radiometer, Copenhagen, Denmark), hematocrit ([hct]_v_ (Na-heparinized Microtubes, B. Braun, Melsungen, Germany), glucose ([glu]_v_) (Biosen 5130, EKF, Barleben, Germany), and lactate [lac]_v_ (Biosen 5030, EKF). The remaining blood was transferred into heparinized tubes containing 20 µL of Liquemin N2500 (Hoffmann-La Roche, Basel, Switzerland) and centrifuged for 10 min at 4 °C and 800× *g* (3–18 K, Sigma, Osterode, Germany) to separate plasma from erythrocytes. The erythrocyte fractions were discarded, and the plasma fractions were aliquoted in 1 mL portions and refrigerated at −80 °C until further processing and analysis. Sample storage conditions are known to influence the analytical outcome of many biochemical parameters, especially including MDA, which can be artificially formed [29,67]. For GC–MS analysis, 1 mL portions samples were thawed in an ice bath once only and used as described below. In the present study, we did not identify any influence of sample storage conditions on the obtained results.

### 4.4. Biochemical GC–MS Analyses in Plasma Samples

Nitrite, nitrate, and creatinine were simultaneously measured using gas chromatography–mass spectrometry (GC–MS) in 100 µL plasma samples as described elsewhere [69] using [^15^N]nitrite (at 0.4 µM), [^15^N]nitrate (at 40 µM), and [methylo-^2^H_3_]creatinine (d_3_-creatinine) (at 100 µM), respectively, with 2,3,4,5,6-pentafluorobenzyl bromide (PFB-Br) as the derivatization reagent. PFB-Br, the sodium salts of [^15^N]nitrite and [^15^N]nitrate (each declared as 99 atom% at ^15^N), and d_3_-creatinine (declared isotopic purity of >99 atom% ^2^H) were obtained from Sigma-Aldrich (Steinheim, Germany). Free amino acids were measured using GC–MS in 10 µL plasma samples as described elsewhere [70] using in situ prepared trideutero-methyl esters of the corresponding amino acids. For methodological reasons, ornithine and citrulline, asparagine and aspartate, and glutamine and glutamate were measured as their sums. Malondialdehyde (MDA), a biomarker of oxidative stress [29], was determined in 100 µL plasma aliquots using [1,3-^2^H_2_]MDA (d_2_-MDA) (at 10 µM) as the internal standard, as described elsewhere [71].

GC–MS analyses were performed on a single-quadrupole mass spectrometer model ISQ directly interfaced with a Trace 1310 series gas chromatograph equipped with an autosampler AS 1310 from ThermoFisher (Dreieich, Germany). The gas chromatograph was equipped with a 15 m long fused silica capillary column Optima 17 (0.25 mm I.D., 0.25 µm film thickness) from Macherey-Nagel (Düren, Germany). The following oven temperature program was used with helium at a constant flow rate of 1 mL/min as the carrier gas: 1 min at 70 °C, then increased to 250 °C and 320 °C at rates of 30 °C/min and 70 °C/min, respectively, and held at 320 °C for 1 min. Interface, injector, and ion source were kept at 300 °C, 280 °C, and 250 °C, respectively. Electron energy was set to 70 eV, and electron current was set to 50 µA in all analyses. These values are recommended by the manufacturer for this GC–MS apparatus. Methane (1.0 mL/min) was used as the reagent gas for negative-ion chemical ionization. Aliquots (1 µL) from toluene extracts were injected in splitless mode by means of the autosampler. Selected ion monitoring (SIM) of the analytes and their stable isotope-labeled analogs as described previously in detail. Each biochemical parameter was measured once only as described elsewhere using fully validated methods. Quality control samples were performed in duplicate. Precision (relative standard deviation, %) and accuracy (recovery, %) for the analytes added to plasma at relevant concentrations [31] were acceptable (below 20% and within the range 80–120%, respectively).

### 4.5. Calculation of Equilibrium Constants

The dimensionless equilibrium constants *K*_harg_ and *K*_gaa_ were calculated as reported elsewhere [30] using the formulas inserted in Scheme 1. The equilibrium constant *K*_harg_ was calculated using the plasma concentrations of the amino acids Arg, Lys, hArg, and Orn/Cit. The equilibrium constant *K*_gaa_ was calculated using the plasma concentrations of Arg, Gly, GAA, and Orn/Cit. The ratio of *K*_harg_ to *K*_gaa_ (i.e., *K*_harg_/*K*_gaa_) was calculated and used for a comparison of both study groups.

### 4.6. Statistical Analyses

Univariate statistical analysis and preparation of graphs including receiver operating characteristic curves (ROC) were performed with GraphPad Prism (version 7 for Windows, La Jolla, CA, USA). Normality was tested by using the D’Agostino and Pearson normality test. Data are reported as the mean ± standard deviation (SD) for normally distributed data and as the median with 95% confidence intervals otherwise. Correlations between parameters were performed after Pearson or Spearman as appropriate. One-way ANOVA and Mann–Whitney test were used for in-group analyses. A two-tailed *p*-value of 0.05 was considered statistically significant.

Multivariate statistical analyses were performed on SAS^®^ OnDemand for Academics and used to describe the relationships among plasma amino-acid concentrations and related parameters such as *K*_gaa_, *K*_harg_, time, and treatment (NaNO_3_ vs. NaCl). Because plasma amino-acid concentrations are largely correlated each other, we used both supervised and unsupervised a posteriori approaches to reduce the data and identify underlying constructs or patterns that best explain the data according to supplementation. In the principal component analysis, we used 25 variables, including amino acids, equilibrium constants, and time. These analyses are described in detail in the Supplementary Materials.

## 5. Conclusions

Short-term inorganic nitrate supplementation increases the hArg synthesis to an extent corresponding to the synthesis capacity of one kidney in healthy humans. The underlying mechanism remains to be elucidated. Differences in the association between plasma concentrations of amino acids in the NaNO_3_ and NaCl groups suggest changes in amino-acid homeostasis. Plasma nitrite concentration is a strong discriminant marker of inorganic nitrate ingestion. Oxidative stress measured as plasma MDA did not change after supplementation of NaNO_3_ or NaCl.

## Figures and Tables

**Table 1:** Plasma concentrations ^a^ of amino acids, creatinine, nitrite, and nitrate and equilibrium constants in the NaCl group (placebo, PL) and NaNO_3_ group (N), their percentage difference (D, %), and the area under the curve (AUC) values of the receiver operating characteristic curves (ROC).

Analyte	NaCl Group (PL)	NaNO_3_ Group (N)	D (%) (N − PL)	*p* ^b^ Value	AUC-ROC
Alanine	477 [414–544]	420 [502–568]	−12	0.3352	0.55
Threonine	143 [114–161]	129 [110–148]	−10	0.0626	0.60
Glycine	250 [234–279]	258 [214–299]	+3	0.4175	0.54
Valine	280 [261–319]	277 [238–326]	−1	0.5982	0.53
Serine	149 [128–175]	144 [128–176]	−3	0.8966	0.51
Sarcosine	1.87 [1.40–2.34]	1.35 [1.11–1.79]	−28	<0.0001	0.73
Leucine/isoleucine	217 [195–252]	215 [197–246]	−1	0.7005	0.52
Guanidinoacetate	3.85 [2.77–4.52]	3.56 [3.09–4.18]	−8	0.7751	0.52
Asparagine/aspartate	69.8 [62.2–76.1]	67.7 [59.4–77.2]	−3	0.4795	0.54
Hydroxy-proline	10.8 [7.26–20.1]	11.3 [9.51–14.0]	+4	0.4870	0.54
Proline	247 [203–290]	253 [236–304]	+2	0.1761	0.57
Methionine	52.2 [44.0–75.1]	51.7 [45.4–84.1]	−1	0.7153	0.52
Glutamine/glutamate	787 [732–855]	836 [800–905]	+6	0.0003	0.69
Ornithine/citrulline	49.8 [46.2–58.0]	59.6 [49.4–67.5]	+16	0.0152	0.63
Phenylalanine	68.9 [62.3–72.2]	63.6 [56.3–69.6]	−8	0.0026	0.66
Tyrosine	69.1 [56.4–78.1]	59.7 [52.7–63.7]	−14	0.0051	0.65
Lysine	201 [147–217]	179 [162–214]	−11	0.2967	0.56
Arginine	98.6 [86.6–112.4]	103 [85.5–114]	+14	0.9796	0.50
Homoarginine	1.27 [1.06–1.68]	1.68 [1.16–1.97]	+24	0.0001	0.70
Tryptophan	44.3 [37.8–51.0]	40.8 [35.4–45.2]	−8	0.0047	0.65
ADMA	0.42 [0.38–0.50]	0.44 [0.38–0.49]	+5	0.8088	0.51
GABR	1.89 [1.63–2.09]	1.81 [1.47–2.10]	−4	0.0921	0.59
Creatinine	118 [97–127]	106 [91–141]	−10	0.4533	0.58
Malondialdehyde	0.319 [0.294–0.415]	0.284 [0.236–0.351]	−10	0.0002	0.693
Potassium	4.63 [4.37–4.86]	4.50 [4.29–4.71]	−3	0.023	0.605
Nitrite	0.88 [0.14–1.06]	1.47 [1.26–1.80]	+40	**<0.0001**	**0.951**
Nitrate	35.5 [26.7–67.7]	263 [170–306]	+640	**<0.0001**	**0.866**
Nitrate/nitrite ratio (P_NOx_)	56.4 [309–203]	130 [50–227]	+57	0.0478	0.605
*K*_gaa_ (×1000)	6.9 [6.1–8.6]	7.7 [6.7–9.4]	+11	0.0334	0.614
*K*_harg_ (×1000)	3.5 [2.8–5.8]	4.9 [4.2–6.6]	+29	<0.0001	0.733
*K*_gaa_/*K*_harg_	1.96 [1.30–3.17]	1.57 [1.31–2.01]	−20	0.0066	0.645

^a^ Plasma concentrations are given as the median [25th–75th percentile] in µM and mM for K^+^. ^b^ Mann–Whitney test was performed. The plasma concentrations measured at all exercise timepoints were considered in both groups. Numbers in bold indicate statistical significance for high AUC-ROC values.

## Data Availability

The study did not report any data.

## Supplementary Materials

The following are available online at https://www.mdpi.com/article/10.3390/ijms231810649/s1.
