# Supplementary material for: Short-Term Supplementation of Sodium Nitrate vs. Sodium Chloride Increases Homoarginine Synthesis in Young Men Independent of Exercise
Source: Int J Mol Sci. 2022 Sep 13;23(18):10649. doi: 10.3390/ijms231810649 (PMC9504822; doi:10.3390/ijms231810649)
Supplement: Supplementary file 1 [file ijms-23-10649-s001.zip › ijms-1857735-supplementary.pdf]

Supplement to

Short-term supplementation of sodium nitrate vs sodium chloride (0.14 mmol/kg/d) increases homoarginine synthesis in young men independent of exercise

Dimitrios Tsikas,[1,a,*] Norbert Maassen,[2,3,a] Antonie Thorns,[2] Armin Finkel,[2,3] Moritz Lützow,[2] Magdalena Aleksandra Röhrig,[2] Larissa Sarah Blau,[2] Laurianne Dimina,[4] François Mariotti,[4] Bibiana Beckmann[1], Vladimir Shushakov,[2,3] Mirja Jantz[2,3,b]

[1] *Institute of Toxicology, Core Unit Proteomics, Hannover Medical School, 30623, Hannover, Germany*

[2] *Institute of Sport Medicine, Hannover Medical School, 30623, Hannover, Germany*

[3] *Institute of Sport Science, Leibniz University Hannover, Hannover, Germany*

[4] *Université Paris-Saclay, AgroParisTech, INRAE, UMR PNCA, 75005, Paris, France*

b Present address: Olympic Training and Service Center of Lower Saxony, Hannover, Germany

(A) Results

Table S1. Baseline plasma concentrations (in µM; K in mM) and nitrate-to-nitrite molar ratio in plasma (P_{NOx}) of the analytes and equilibrium constants K_{harg}, K_{gaa} and K_{gaa}/K_{harg} in the subjects of the study groups. Data are reported as median with interquartile range or as mean with standard deviation. Mann-Whitney test was performed.

Analytes	NaCl group (*n* = 8)	$NaNO_3$ group (*n* = 9)	*P* value
Nitrite	2.2 [1.93-2.29]	2.48 [2.11-2.55]	0.319
Nitrate	46.5 [42.8-55.0]	43.9 [37.6-52.5]	0.913
Nitrate/Nitrite (P_{NOx})	21.7 [18.5-26.5]	19 [15.7-20.9]	0.209
Creatinine	92 [84-101]	87.5 [81.6-96.5]	0.497
Malondialdehyde	0.37 [0.31-0.42]	0.39 [0.31-0.46]	0.747
Kalium (mM)	4.15 ± 0.41	3.92 ± 0.21	0.154
Alanine	355 [260-392]	323 [254-379]	0.518
Threonine	130 [107-162]	112 [105-147]	0.421
Glycine	213 [176-248]	197 [147-233]	0.501
Valine	232 [220-286]	253 [218-265]	0.386
Serine	128 [106-210]	128 [124-139]	0.562
**Sarcosine**	**2.24 [2.0-2.58]**	**1.34 [1.27-1.84]**	**0.005**
Leucine/Isoleucine	181 [160-254]	189 [155-220]	0.326
Guanidinoacetate	2.29 [1.77-2.92]	2.28 [1.84-4.57]	0.835
Aspartate/Asparagine	64.9 [56.4-94.0]	59.5 [48-74]	0.165
Hydroxy-proline	8.88 [5.77-10.9]	8.55 [6.26-10.7]	0.962
Proline	165 [118-230]	184 [152-204]	0.878
Methionine	52.6 [44.5-58.5]	47.9 [40.8-55-5]	0.362
Glutamate/Glutamine	617 [472-651]	581 [449-654]	0.662
Ornithine/Citrulline	38.4 [34.1-45.8]	42.9 [33.7-47.6]	0.530
Phenylalanine	55.7 [42.2-69.0]	53.5 [39.4-61.6]	0.371
Tyrosine	44.5 [43.1-63.9]	43.5 [36.2-52.6]	0.272
Lysine	149 [130-171]	136 [110-152]	0.131
Arginine	75.6 [50.4-89.1]	72.5 [55.6-90.2]	0.946
Homoarginine	1.30 [0.66-1.45]	1.49 [0.88-1.74]	0.325
Tryptophan	14.2 [10.8-20.4]	14.3 [6.31-19.2]	0.439
ADMA	0.380 [0.315-0.534]	0.317 [0.187-0.460]	0.277
K_{gaa} (×1000)	5.69 [4.41-6.98]	8.76 [5.23-10.2]	0.179
K_{harg} (×1000)	4.24 [2.74-5.45	5.67 [4.81-6.68]	0.132
K_{gaa}/K_{harg}	1.56 [1.01-1.90]	1.30 [1.05-1.87]	0.818

Table S2. Number of statistically significant Spearman correlations at baseline between the plasma concentrations of the listed amino acids in the subjects of the two study groups

Amino acid	*Number of correlations ($P < 0.05$)*
Alanine	11
Threonine	14
Glycine	12
Valine	10
Serine	6
Sarcosine	0
Leucine/Isoleucine	9
Guanidinoacetate	3
Aspartate/Asparagine	10
Hydroxy-proline	7
Proline	5
Methionine	6
Glutamate/Glutamine	6
Ornithine/Citrulline	4
Phenylalanine	3
Tyrosine	3
Lysine	1
Arginine	1
Homoarginine	0
Tryptophan	1
ADMA	0

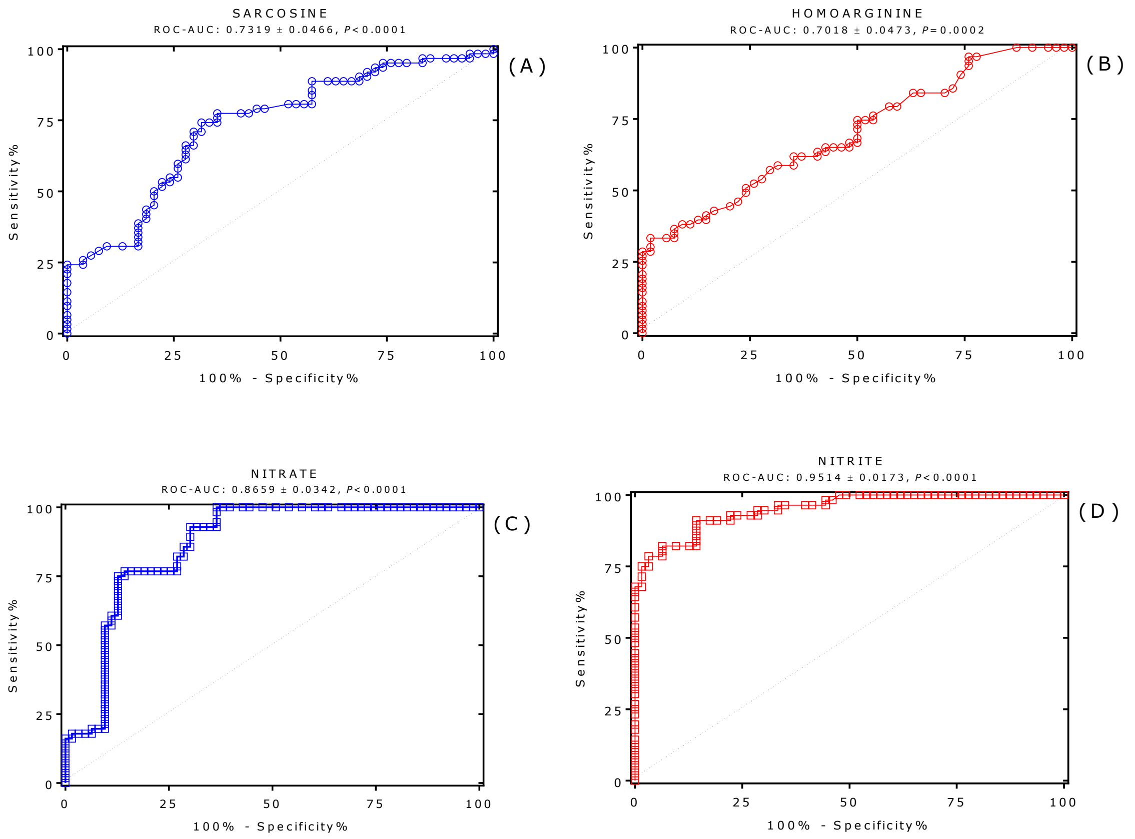

Figure S1. Area under the curve (AUC) of receiver operation characteristic (ROC) of (A) plasma sarcosine, (B) plasma homoarginine, (C) plasma nitrate and (D) plasma nitrite after supplementation of NaCl (*n* = 63) or $NaNO_3$ (*n* = 56). All concentrations measured during the seven exercise steps of all volunteers were included (see Scheme 2).

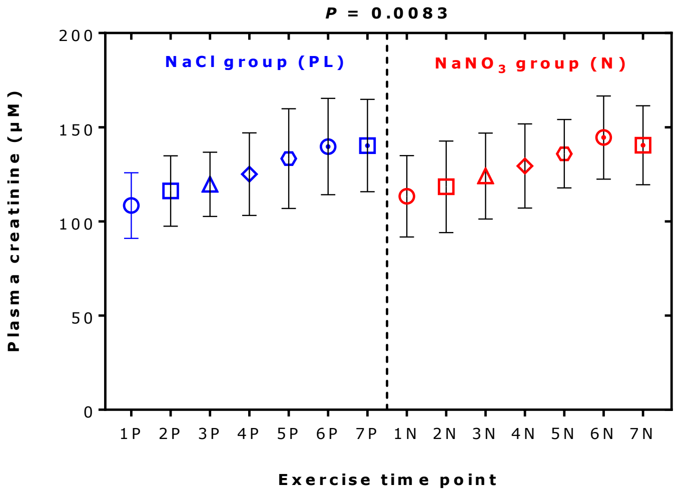

Figure S2. Plasma creatinine concentrations in the volunteers of the NaCl group (PL) and the $NaNO_3$ group (N) at the seven exercise time points after supplementation. Data are shown as mean with standard error of the mean. One-way ANOVA, $P = 0.0083$.

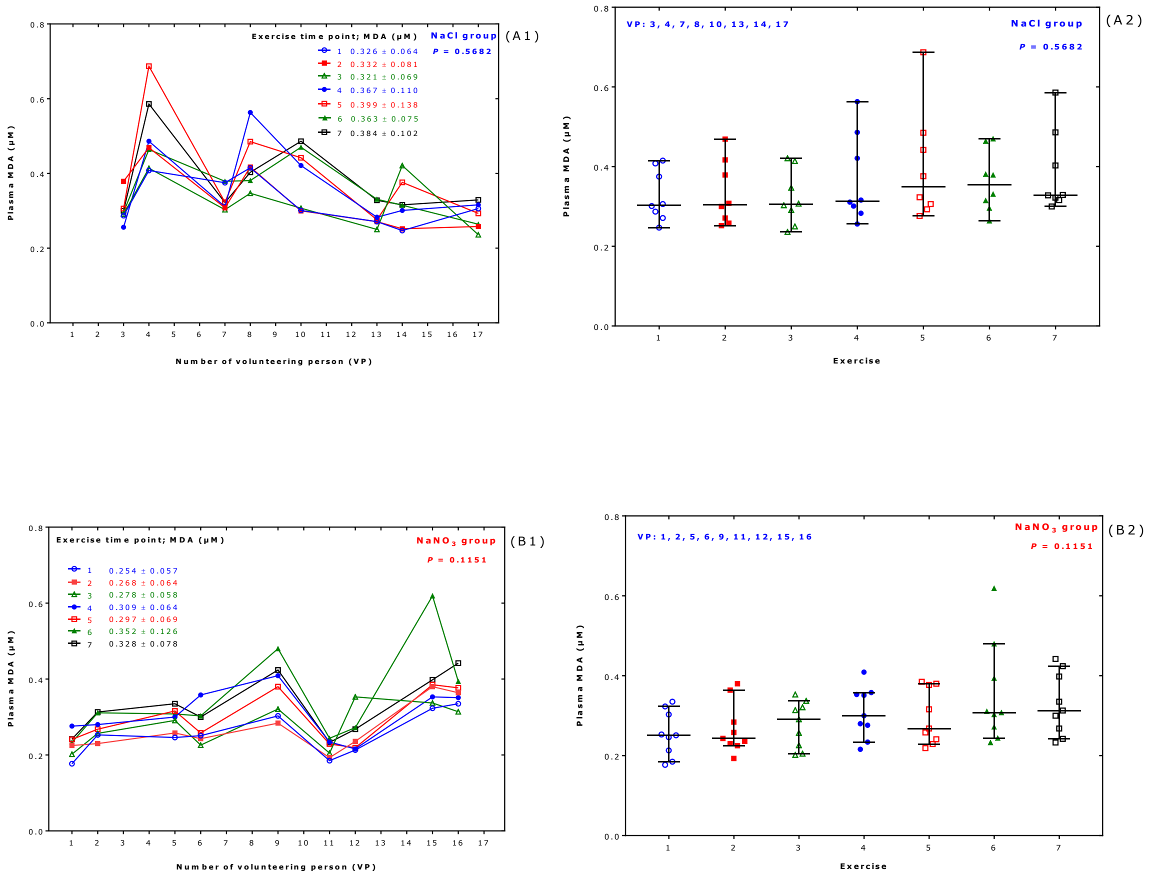

Figure S3. Plasma malondialdehyde (MDA) concentrations in the volunteering persons (VP) of the NaCl (A1, A2) and $NaNO_3$ (B1, B2) groups at the seven individual time points of exercise after supplementation (see Scheme 2). Data in (A2) and (B2) are shown as median with 95% confidence interval.

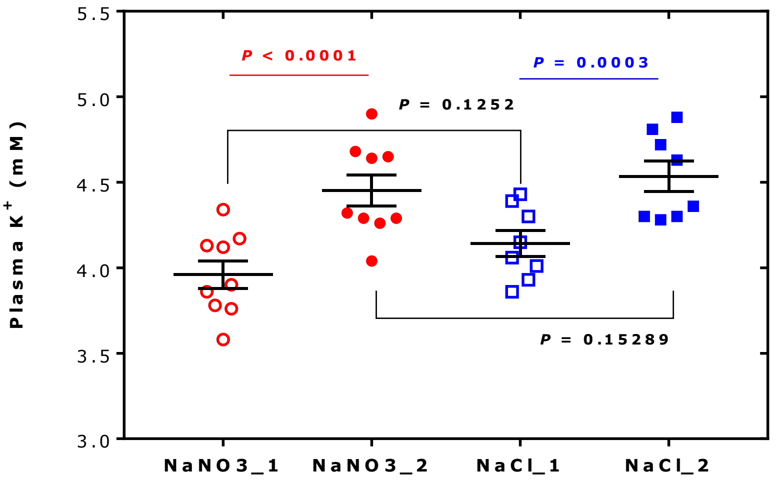

Figure S4. Plasma kalium (K^+) concentrations in the volunteering persons (VP) of the NaCl and $NaNO_3$ groups at exercise 1 and 2 after supplementation. Data are shown as mean with standard error of the mean.

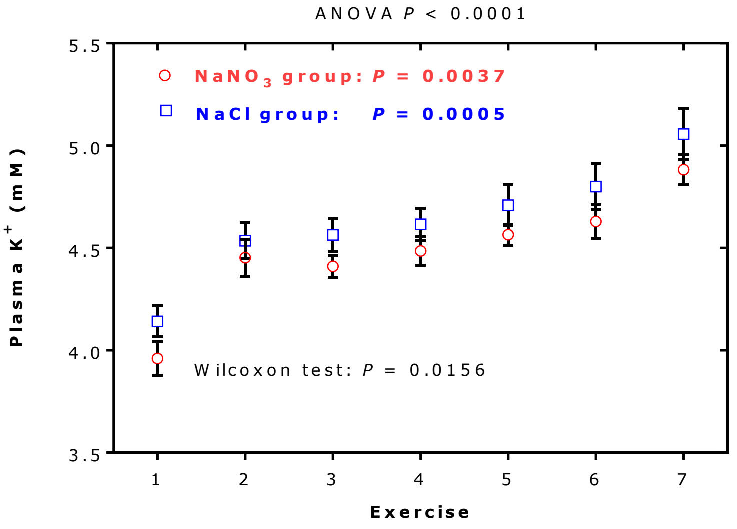

Figure S5. Plasma kalium (K^+) concentrations in the volunteering persons of the NaCl and $NaNO_3$ groups at the seven exercise time points after supplementation of NaCl or $NaNO_3$ (see Scheme 2). Data are shown as mean with standard error of the mean.

Figure S6 (Panel A to X). Spearman coefficients of correlation between an analyte vs. each of the other analytes in the $NaNO_3$ and NaCl groups after supplementation. The data of all exercise time points were considered. Only statistically significant correlations (r > 0.25, P<0.05) are plotted. The numbers within the pictures give the number of correlations. Each symbol indicates an analyte. Symbols connected by a line indicate the same analyte. GABR, global arginine bioavailability.

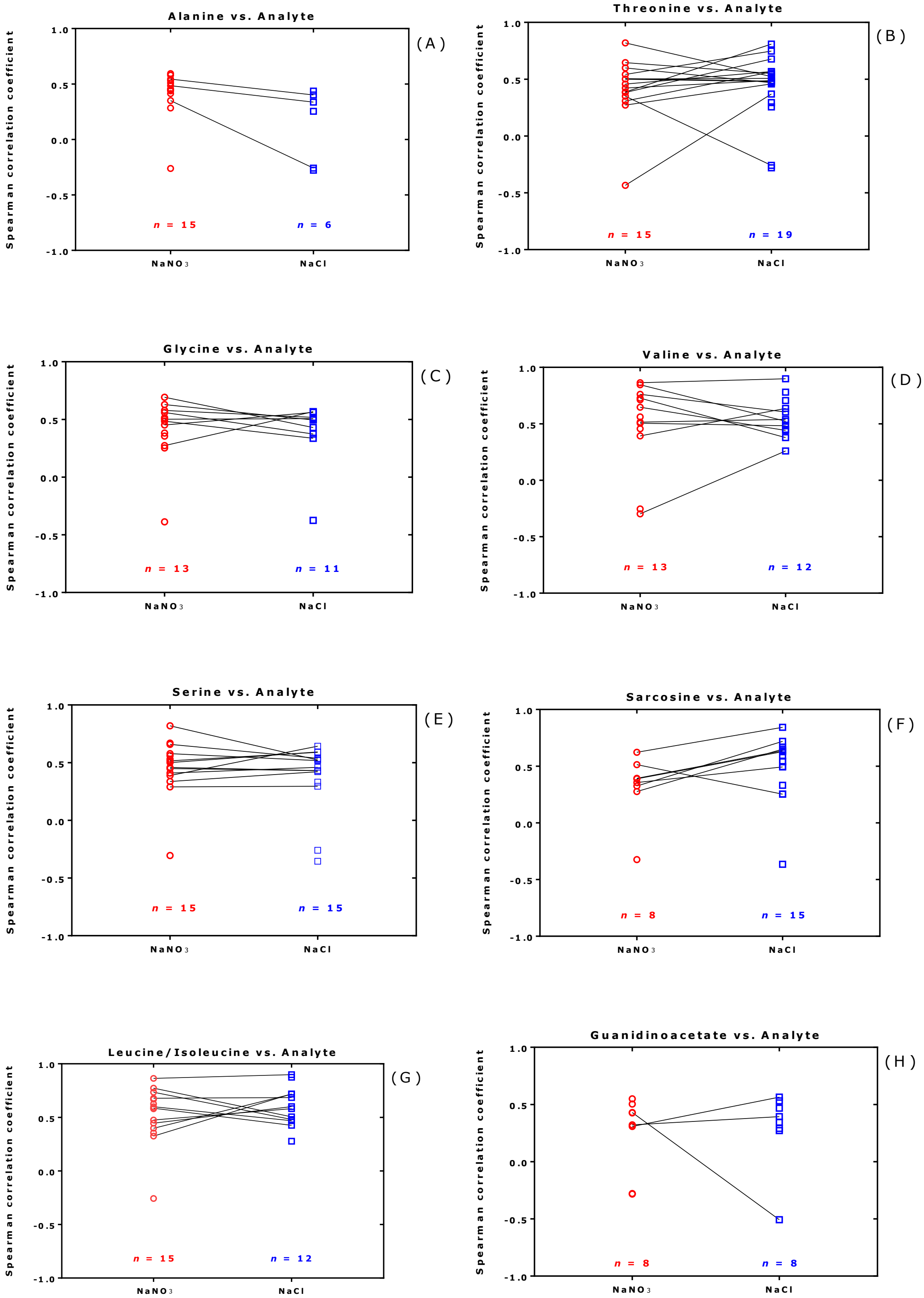

Figure S6 (Panel A to X) *continued*

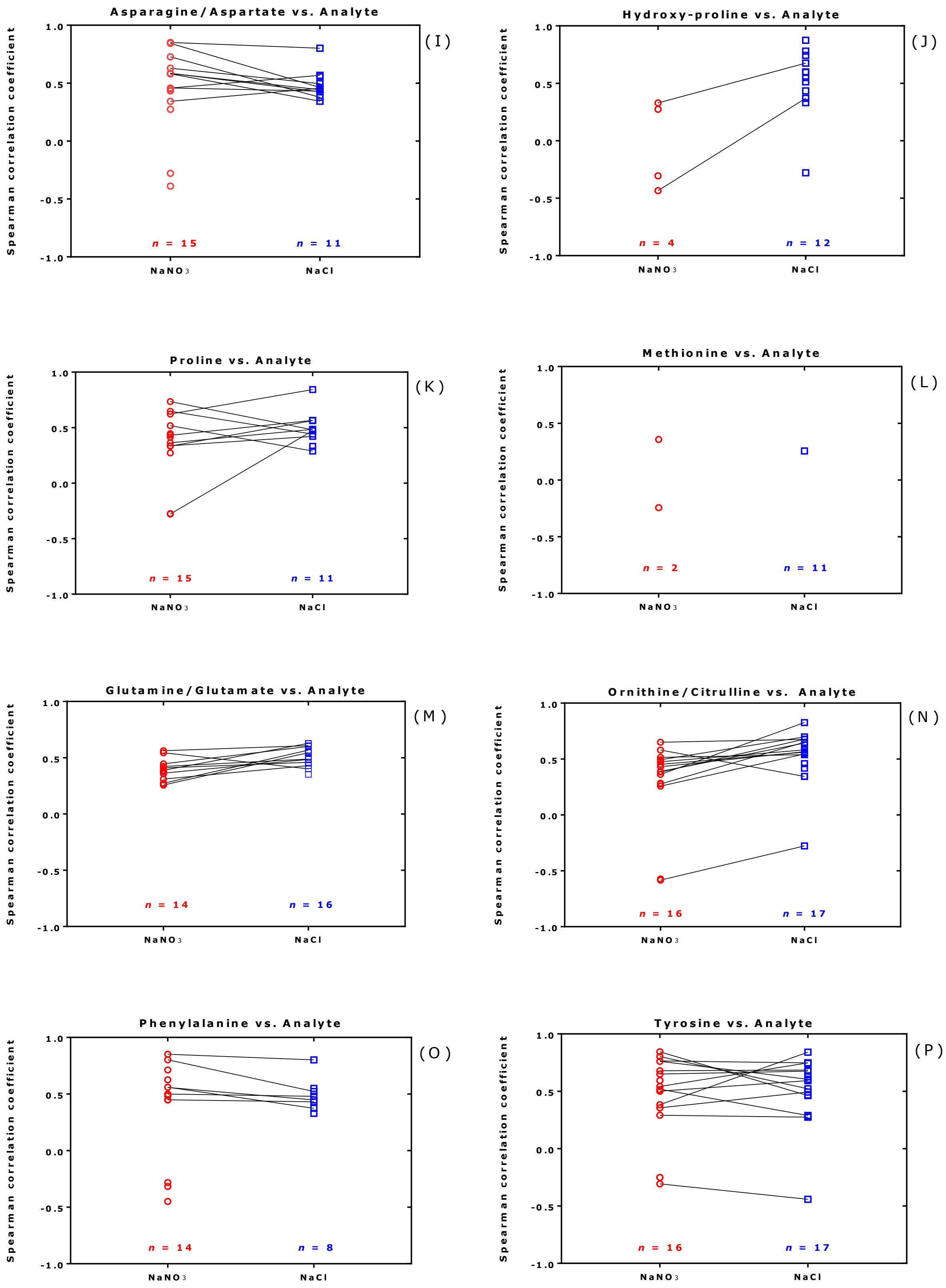

Figure S6 (Panel A to X) *continued*

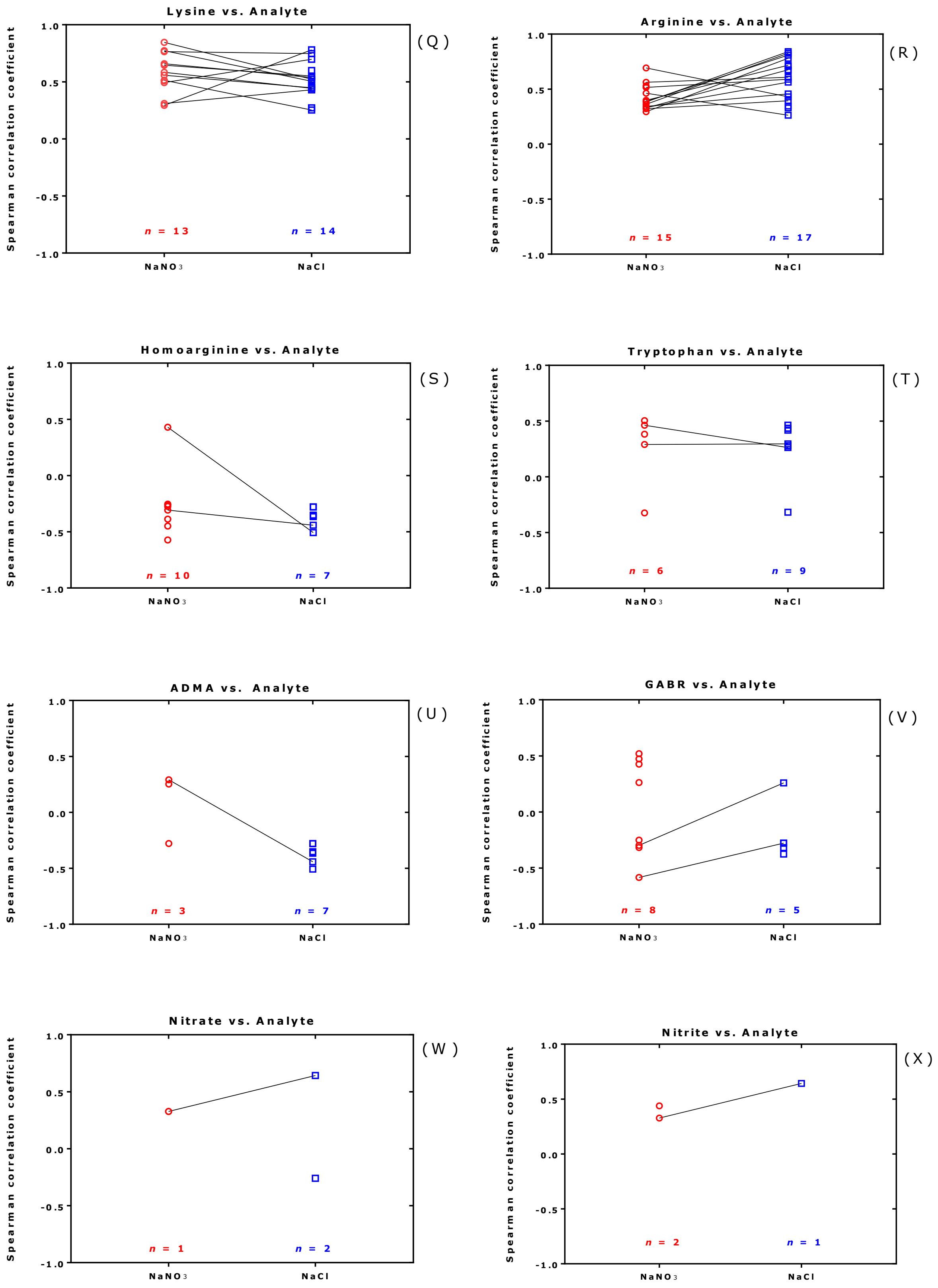

(B) Multivariate statistical analyses

Multivariate statistical analyses were performed on SAS® OnDemand for Academics and used to describe the relationships among plasma amino acids concentrations, and related parameters such as K_{gaa}, K_{harg}, time and kind of treatment (NaCl vs $NaNO_3$). Because plasma amino acids concentrations are largely correlated each other, we used both supervised and unsupervised *a posteriori* approaches to reduce the data and identify underlying constructs or patterns that best explain data according to supplementation. In the analysis, we used 25 variables, including amino acids, equilibrium constants and time.. Multivariate statistical analyses were performed on SAS® OnDemand for Academics. These analyses are described in detail in the Supplement to this work

1) Unsupervised PCA

Principal Component Analysis (PCA) was applied on the 25 standardized variables. Three components were extracted, explaining 57% of the total variance (Figure S7).

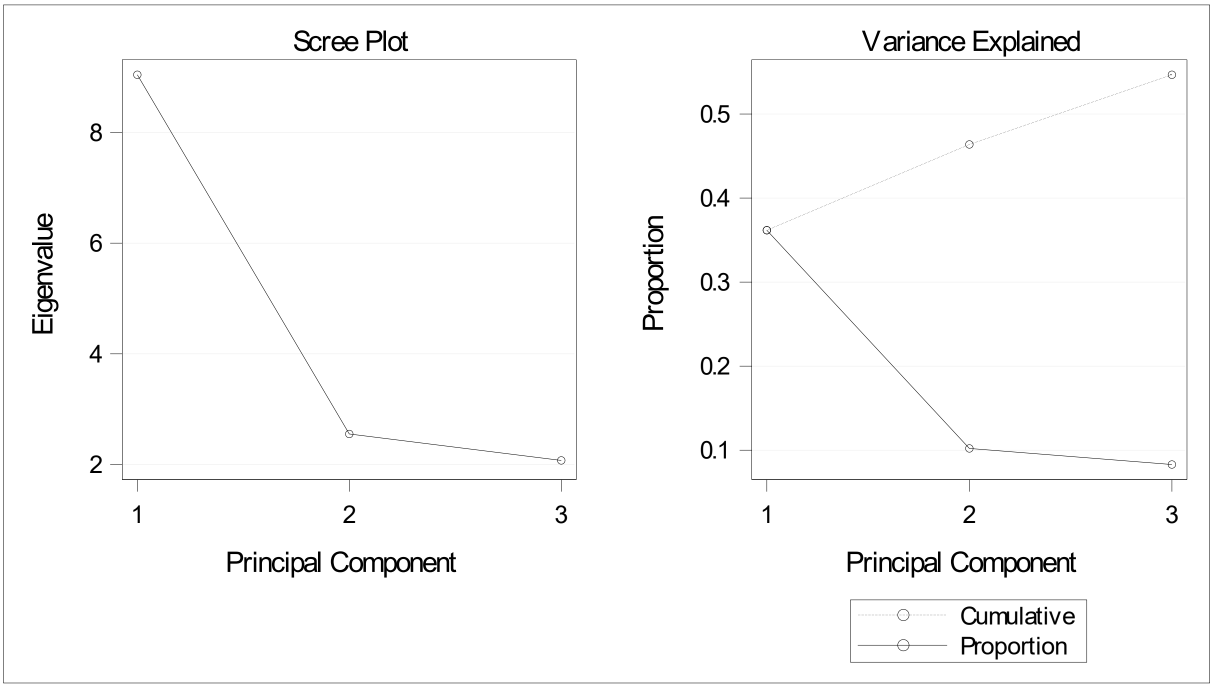

Figure S7. Scree plots of Eigenvalues and proportion of variance explained by the top three principal components.

Orthogonal rotation of the components was undertaken using the varimax procedure. The variable loadings of the components are shown in Table S3.

Table S3. Correlations of variables with the top three Eigenvectors

Variables	Eigenvectors		
	V1	V2	V3
**Time (min)**	-0.003464	0.114890	0.129442
**Ala**	0.146228	-0.004953	0.325103
**Thr**	0.257117	0.125367	-0.023161
**Gly**	0.210201	-0.081152	0.151410
**Val**	0.255698	-0.193970	-0.012837
**Ser**	0.241854	0.146185	0.121267
**Sarcosine**	0.208889	-0.039877	0.064527
**Leu/Ile**	0.283600	-0.155690	0.066761
**GAA**	0.113697	0.512763	0.077623
**Asp/Asn**	0.246950	-0.233925	-0.091398
**OH-Pro**	0.156428	-0.148923	-0.042922
**Pro**	0.208688	-0.113759	0.193511
**Met**	0.031089	0.235720	0.172185
**Glu/Gln**	0.179184	0.114400	0.427445
**Orn/Cit**	0.228001	0.029496	0.088742
**Phe**	0.221147	-0.136237	-0.226708
**Tyr**	0.297981	-0.005661	-0.165702
**Lys**	0.255571	0.012233	-0.079437
**Arg**	0.264239	-0.042029	0.150376
**hArg**	-0.129028	-0.060647	0.416006
**Trp**	0.113324	0.144607	-0.011981
**ADMA**	0.089594	0.147920	0.191116
K_{gaa}	-0.021955	0.482388	-0.009537
K_{harg}	-0.234933	-0.053022	0.313777
K_{gaa}/K_{harg}	0.165389	0.393541	-0.375029

2) Supervised PLS

Partial least squares (PLS) regression was used with 25 variables as response factor and treatment as categorical predictor using NIPALS algorithm. We rather focused on finding a

few variables that contribute most to separating the data between the two-predictor modalities.

PCA

The C1/C3 factorial plan best discriminates the effect of $NaNO_3$ vs NaCl. Five variables are well represented on this picture and suggest that $NaNO_3$ supplementation results in higher plasma hArg and a higher K_{harg} equilibrium constant, while NaCl supplementation results in higher plasma aromatic amino acids and in a higher K_{gaa}/K_{harg} ratio. See Figure 7 in the main text.

PLS

In the model, 100% of the response variation is explained by just one factor but only explains 4.7% of the predictor variation. The coefficients for predicting the responses were used as a tool for finding a few variables with the biggest coefficient that contribute most to separating the data between the two modalities of the treatment ($NaNO_3$ and NaCl). Glu+Gln, hArg plasma concentrations and K_{gaa} and K_{harg} are the best single contributor to predict NaN03 treatment, whereas sarcosine, tryptophan, phenylalanine, and tyrosine plasma concentrations and the K_{gaa}/K_{harg} ratio better contribute to predict NaCl treatment (Figure S8, Table S3).

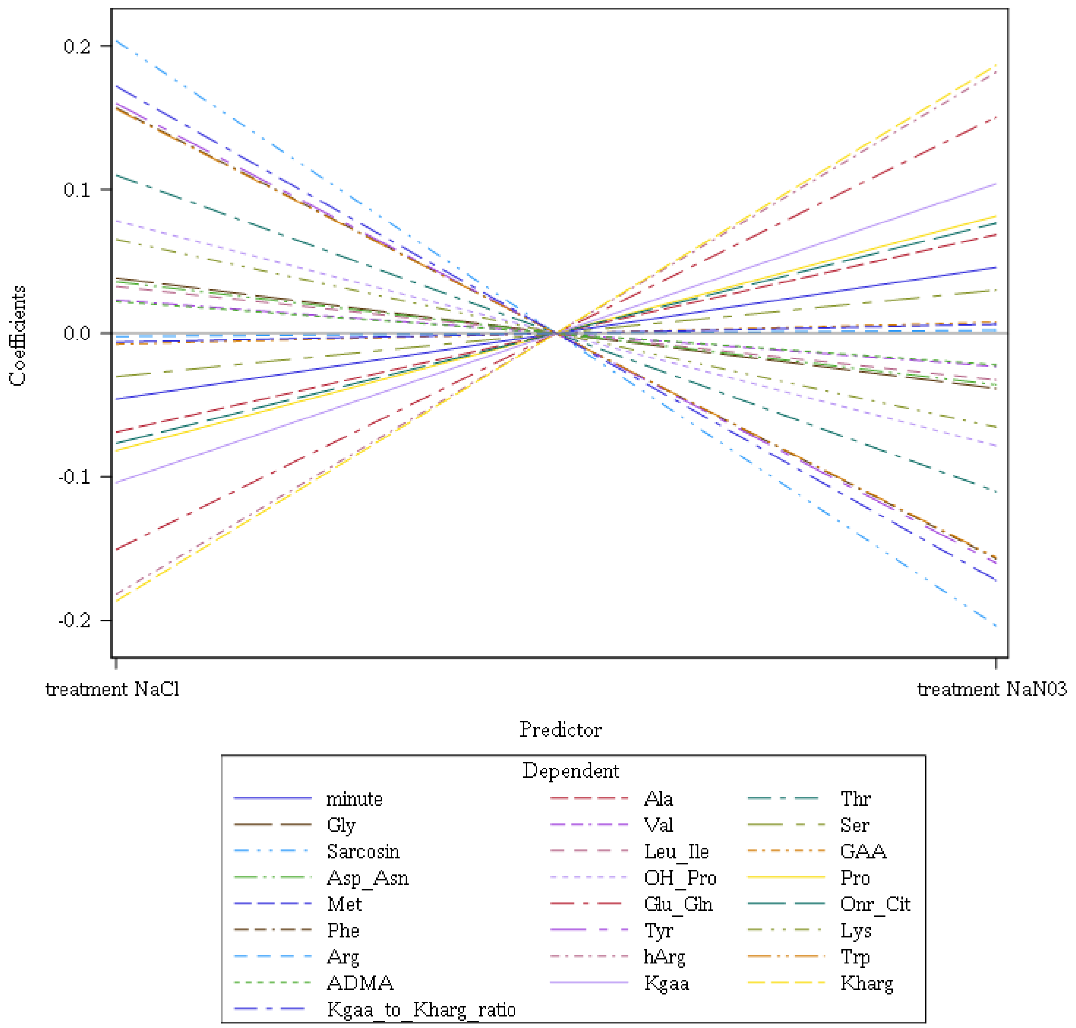

Figure S8: Profiles of centered and scaled parameter estimates according to treatment modality.

Table S4. Coefficient estimates of the final predictive model for the responses variables (centered and scaled data). Intercept value is zero (not shown)

	**Variable**	**$NaNO_3$ vs NaCl**
**Parameter Estimates for Centered and Scaled Data**	Time (minute)	+0.05
	Ala	+0.07
	Thr	-0.11
	Gly	-0.04
	Val	-0.02
	Ser	+0.03
	**Sarcosine**	**-0.20**
	Leu/Ile	-0.03
	GAA	+0.01
	Asp/Asn	-0.04
	OH-Pro	-0.08
	Pro	+0.08
	Met	+0.01
	**Glu/Gln**	**+0.15**
	Orn/Cit	+0.08
	**Phe**	**-0.16**
	**Tyr**	**-0.16**
	Lys	-0.07
	Arg	+0.00
	**hArg**	**+0.18**
	**Trp**	**-0.16**
	ADMA	-0.02
	**K_{gaa}**	**+0.10**
	**K_{harg}**	**+0.19**
	**K_{gaa}/K_{harg}**	**-0.17**
